# Exogenous loading of miRNAs into small extracellular vesicles

**DOI:** 10.1002/jev2.12111

**Published:** 2021-08-02

**Authors:** Ricardo C. de Abreu, Cristiana V. Ramos, Clarissa Becher, Miguel Lino, Carlos Jesus, Paula A. da Costa Martins, Patrícia A. T. Martins, Maria João Moreno, Hugo Fernandes, Lino Ferreira

**Affiliations:** ^1^ CNC ‐ Centro de Neurociências e Biologia Celular CIBB ‐ Centro de Inovação em Biomedicina e Biotecnologia University of Coimbra Coimbra Portugal; ^2^ Faculty of Health, Medicine and Life Sciences CARIM School for Cardiovascular Diseases Maastricht University Maastricht The Netherlands; ^3^ Department of Molecular Genetics Faculty of Sciences and Engineering Maastricht University Maastricht The Netherlands; ^4^ Chemistry Department Faculty of Science and Technology Coimbra Chemistry Centre University of Coimbra Coimbra Portugal; ^5^ Faculty of Medicine University of Coimbra Coimbra Portugal

**Keywords:** extracellular vesicles, microRNA, modulation, post‐isolation

## Abstract

Small extracellular vesicles (sEVs), through their natural ability to interact with biological membranes and exploit endogenous processing pathways to convey biological information, are quintessential for the delivery of therapeutically relevant compounds, such as microRNAs (miRNAs) and proteins. Here, we used a fluorescently‐labelled miRNA to quantify the efficiency of different methods to modulate the cargo of sEVs. Our results showed that, compared with electroporation, heat shock, permeation by a detergent‐based compound (saponin) or cholesterol‐modification of the miRNA, Exo‐Fect was the most efficient method with > 50% transfection efficiency. Furthermore, qRT‐PCR data showed that, compared with native sEVs, Exo‐Fect modulation led to a > 1000‐fold upregulation of the miRNA of interest. Importantly, this upregulation was observed for sEVs isolated from multiple sources. The modulated sEVs were able to delivery miR‐155‐5p into a reporter cell line, confirming the successful delivery of the miRNA to the target cell and, more importantly, its functionality. Finally, we showed that the membrane of Exo‐Fect‐loaded sEVs was altered compared with native sEVs and that enhanced the internalization of Exo‐Fect‐loaded sEVs within the target cells and decreased the interaction of those modulated sEVs with lysosomes.

## 1 INTRODUCTION

Extracellular vesicles (EVs) are biological particles secreted by most organisms and cell types (Raposo & Stoorvogel, 2013). In recent years, particular attention has been given to small EVs (sEVs), vesicles with a diameter between 30–200 nm capable of permeating biological barriers and deliver their cargo onto target cells (Van Niel et al., 2018). There is an increasing interest to use these vesicles as vehicles for the delivery of biomolecules such as miRNAs, short (∼22 nucleotides) non‐coding nucleic acids that regulate gene expression at the post‐transcriptional level, for the treatment of cardiovascular, neurodegenerative diseases, among others.

Early attempts to modulate the content of sEVs focused on modifications to the secreting cell such as, for example, transfection with the gene of interest or addition of small molecules to the culture medium (Hung & Leonard, 2016; Kanada et al., 2015; Montecalvo et al., 2012). This approach remains the most widely used strategy to enrich or deplete sEVs of any molecule of interest. However, this methodology is not applicable to sEVs isolated from biological fluids. Moreover, the establishment of in vitro cell cultures dedicated to sEV production is time consuming and costly. Therefore, the post‐isolation modification of sEVs with exogenous biomolecules of interest has been investigated in recent years. Strategies used for the transfection of cells, such as electroporation (Alvarez‐Erviti et al., 2011; Momen‐Heravi et al., 2014; Wahlgren et al., 2012), heat shock (Zhang et al., 2016) and detergent‐based (Fuhrmann et al., 2015) permeabilization of the membrane, were used for the modulation of sEVs. The results obtained indicated that small RNAs could be successfully introduced into sEVs and the modulated sEVs were capable of delivering their cargo to the target cell ultimately regulating their function. These results laid the groundwork for the modification of EVs after their purification. Yet, a direct comparison between the different methods of miRNA loading into sEVs has not been performed and, more importantly, several important questions remain unanswered such as, for example, whether the loaded molecule is in the lumen and/or at the membrane of sEVs and whether modulation of sEVs affects their biophysical properties and ultimately their intracellular trafficking properties and capacity to deliver the cargo.

In this work we compared, side‐by‐side, five different methodologies to load miRNAs into sEVs isolated from three different sources: (i) conditioned medium from human umbilical cord blood derived mononuclear cells (hUCBMNCs), (ii) human urine and (iii) commercially available foetal bovine serum. The methodologies tested were based in sEV electroporation (Alvarez‐Erviti et al., 2011; Momen‐Heravi et al., 2014), heat shock in the presence of calcium chloride (Zhang et al., 2016), saponin permeabilization (Fuhrmann et al., 2015), conjugation of the miRNA with cholesterol (O'loughlin et al., 2017) and transfection with the commercial kit Exo‐Fect (Lee et al., 2016; Pi et al., 2018). Firstly, the methodologies were ranked based on their effectiveness in loading a fluorescently labelled miRNA into sEVs, Exo‐Fect being the most effective. Then, the selected method was compared with the transfection of the donor cell – used for the enrichment of miRNA in sEVs. Finally, the biophysical properties of Exo‐Fect‐modulated sEVs, namely their size, zeta potential, membrane permeation, cytotoxicity, internalization and intracellular trafficking were characterized and the activity of the loaded miRNA was validated in a reporter cell line. Our results indicated that the loading of miRNAs with Exo‐Fect was the most promising approach to modulate the content of sEVs and that upon modulation, sEVs retained their capacity to efficiently deliver their cargo into recipient cells. Additionally, compared to their native counterparts, Exo‐Fect‐modulated sEVs showed decreased colocalization with lysosomal and early endosomal compartments.

## 2 MATERIALS AND METHODS

### 2.1 sEV collection via differential ultracentrifugation

All human umbilical cord blood (hUCB) samples were obtained upon signed informed consent, in compliance with Portuguese legislation. The collection was approved by the ethical committee of Centro Hospitalar e Universitário de Coimbra, Portugal (HUC‐01‐11). The samples were stored and transported to the laboratory in sterile bags with anticoagulant solution (citrate‐phosphate‐dextrose) and processed within 48 h after collection as previously described by us (Banerjee et al., 2019; Henriques‐Antunes et al., 2019). Briefly, mononuclear cells (MNCs) were isolated by density gradient separation (Lymphoprep ‐ StemCell Technologies SARL, Grenoble, France). To obtain MNC‐derived sEVs (mEVs), hUCB MNCs were cultured in X‐VIVO 15 serum‐free cell culture medium (Lonza) supplemented with Flt‐3 (100 ng/ml, PeproTech) and stem‐cell factor (100 ng/ml, PeproTech) under hypoxia (0.5% O_2_) conditions for 18 h. Conditioned medium was collected and centrifuged at 300 g, for 10 min, at 4°C to remove cells followed by a centrifugation at 2.000 g, for 20 min, at 4°C to deplete cellular debris.

To obtain human urine‐derived sEVs (uEVs), the first morning midstream urine was collected from healthy donors upon signed informed consent and upon approval from the Ethics Committee of the Faculty of Medicine, University of Coimbra (CE‐070‐2019). Samples were centrifuged at 2.000 g, for 20 min, at 4°C to pellet cells and cell debris. After centrifugation, the supernatant was collected, diluted 1:3 with Tris‐EDTA (20 mM, pH 9.0) and vortexed 90 s at 2.500 rpm to disrupt aggregates.

To obtain FBS‐derived sEVs (fEVs), commercial FBS (#10270106, Gibco) was thawed slowly at room temperature (RT) and diluted 1:4 in phosphate buffered saline (PBS).

Wharton‐Jelly derived mesenchymal stromal cells (WJ‐MSCs) were kindly donated by Crioestaminal. Cells were cultured at 5000 cells/cm^2^ in MEM Alpha modification, with L‐glutamine, ribo‐ and deoxyribonucleosides (SH30265, GE Healthcare) supplemented with 10% (v/v) sEV‐depleted FBS (FBS was depleted of sEVs by ultracentrifugation at 100.000 g, for 18 h, at 4°C) and 0.5% (v/v) penicillin/streptomycin (P/S) for 24 h. Subsequently, WJ‐MSCs were transfected with 25 nM of miR‐155‐5p (for some experiments miR‐155‐5p was labelled with Cy3 at the 3′ of the passenger strand) using Lipofectamine RNAimax according manufacturer´s instructions. Non‐transfected WJ‐MSCs were used as a control. After 24 h of transfection, the transfection medium was discarded and WJ‐MSCs were cultured on α‐MEM supplemented with 10% (v/v) sEV‐depleted FBS for further 48 h. Conditioned medium was collected and centrifuged at 300 g, for 10 min, at 4°C to remove cells followed by a centrifugation at 2.000 g, for 20 min, at 4°C to deplete cellular debris.

Regardless of the source, sEVs were purified by differential centrifugation as described previously (Théry et al., 2006). Briefly, samples were ultracentrifuged twice at 10.000 g, for 30 min, at 4°C, the pellet was discarded and the supernatant was submitted to an ultracentrifugation at 100.000 g, for 2 h, at 4°C, to pellet sEVs. Finally, the pellet from the last step was washed with cold PBS, ultracentrifuged again at 100.000 g, for 2 h, at 4°C, resuspended in 150 μl of cold PBS and stored at ‐80°C. Ultracentrifugation steps were performed using a swinging bucket rotor SW 32 Ti in an Optima XPN 100K ultracentrifuge (Beckman Coulter, California, U.S.A.) and 28.7 ml polyallomer conical tubes (Beckman Coulter).

### 2.2 sEV purification via OptiPrep Density Gradient (ODG)

Native and modulated sEVs were purified using ODG according to standard protocols, described previously (Van Deun et al., 2014). Briefly, discontinuous gradient solutions with 5%, 10%, 20% and 40% iodixanol were prepared by mixing a working buffer [0.25 M sucrose, 6 mM EDTA, 60 mM Tris‐HCl, (pH 7.4)], a homogenization buffer [0.25 M sucrose, 1 mM EDTA, 10 mM Tris‐HCL, (pH 7.4)] and a stock solution of OptiPrep ([60% (w/v) aqueous iodixanol solution], in appropriate proportions. Specifically, to prepare the gradient, Optiprep was diluted 5:1 with working buffer to obtain a 50% Optiprep solution, hereafter denoted working solution. Then, 40%, 20%, 10% and 5% gradients were prepared by mixing 4, 2, 1 and 1 parts of working solution with, respectively, 1, 3, 4 and 9 parts of homogenization buffer. In a UC polyallomer tube, 6 ml of 10%, 20% and 40% solutions and 5 ml of the 5% solution were layered on top of each other in decreasing concentrations of iodixanol and subsequently 1 ml of sEV sample was carefully layered on top of the gradient. Preparations were ultracentrifuged at 100.000 g, for 18 h, at 4°C upon which 15 fractions of around 1.5 ml were collected and further analysed. Ultracentrifugation steps were performed using a swinging bucket rotor SW 32 Ti in an Optima XPN 100K ultracentrifuge (Beckman Coulter, California, U.S.A.) and 28.7 ml polyallomer conical tubes (Beckman Coulter).

### 2.3 sEV characterization by nanoparticle tracking analysis (NTA)

Size and concentration of sEVs was performed through NTA using the NanoSight NS300 (Malvern Instruments, Malvern, U.K.). The system used an O‐Ring Top Plate and the sample was injected manually at an approximate flow of 1 ml every 20 s. sEVs were diluted in PBS until a concentration between 15 and 45 particles/frame was reached. For each sample, 5 videos of 30 s were recorded with the camera level set at 16. All the videos were processed with NTA 3.2 analytical software, using the software threshold between 2 and 4 depending on the quality of the videos.

### 2.4 sEV characterization by protein quantification

sEV protein quantification was performed using the microBCA protein assay kit (Thermo Fisher Scientific, Massachusetts, U.S.A.), as per the manufacturer's instructions. Briefly, bovine serum albumin (BSA) was used to obtain a 10 points standard curve. Then, sEV samples were diluted 22 times in 2% (v/v) sodium dodecyl sulphate (SDS) to disrupt the sEV membrane and subsequently, 50 μl of the previous mix was pipetted, in duplicate, into a 96‐well Corning Costar cell culture plates (Corning Inc., New York, U.S.A.). Reaction solution provided in the kit was added and incubated for 2 h at 37°C. Next, the plates were equilibrated at room temperature for 15 min and finally, the absorbance at 562 nm was read in the microplate reader Synergy™ H1 (Biotek, Vermont, U.S.A.).

### 2.5 Western blot analysis

Western blot analysis for the detection of EV markers and contaminants was performed. Briefly, up to 15 μl of concentrated EV preparations in PBS (0.5 to 4 μg) were mixed with 5 μl 4x Laemmli buffer (0.25 M Tris base, 8% SDS, 40% glycerol, 200 mg bromophenol blue, 10% 2‐mercaptoethanol) and boiled at 96°C for 10 min. For the analysis of tetraspanins, Laemmli buffer was prepared without reducing agents. Samples were loaded in 30 μl wells, Any kD Mini‐PROTEAN TGX Stain‐Free Protein Gel (Bio‐Rad # 4568123) and gel electrophoresis was performed in 1 × Tris/Glycine/SDS buffer prepared from a commercial 10 × concentrated stock (10 × Tris/Glycine/SDS Electrophoresis Buffer; Bio‐Rad #1610772), at the constant voltage of 120V, for 75 min. Afterwards, gels were placed in blotting buffer (25 mM Tris, 192 mM glycine, 20% methanol in water) for 10 min to equilibrate. Then the gel was stacked on top of a nitrocellulose membrane (GE Healthcare #10600016) and both were assembled within a transfer system. Transfer was performed in wet conditions at 200 mA for 90 min. Afterwards, the membrane was removed and blocked in a 1:1 PBS‐Tween 20 (0.2% (v/v)) with Intercept Blocking Buffer (Li‐cor #927‐70001) solution for 1 h at room temperature. Membranes were then washed with PBS‐Tween 20 and left to incubate overnight at 4°C with the appropriate primary antibodies and according to the manufacturer recommendation (antibody details below). Then, membranes were washed 3 times with PBS‐Tween and incubated for 1 h at room temperature with secondary antibodies. Membranes were then washed 3 times and viewed in the Odyssey CLx system (Li‐cor) at the 700 nm and 800 nm wavelengths. Antibodies used in this study were: CD63 (BD Pharmingen #556019), ApoA‐1 (Santa Cruz #sc‐376818), GAPDH (Millipore, MAB374), Calnexin (Santa Cruz #sc‐23954), Alix (Cell Signaling, #2171S), CD9 (BD Pharmingen #555370), THP (Santa Cruz #sc‐271022) and IRDye® 800CW Goat anti‐Mouse IgG Secondary Antibody (Li‐cor #926‐32210).

### 2.6 sEV characterization by transmission electron microscopy (TEM)

TEM analyses of sEVs were performed as previously described (Théry et al., 2006). Briefly, samples were diluted 1:1 in 4% (v/v) paraformaldehyde (PFA) and placed on Formvar‐carbon coated grids (TAAB Technologies) for 20 min at RT. After washing 4 times with PBS, grids were placed on a drop of 1% (v/v) glutaraldehyde for 5 min, followed by 5 washes with distilled water, 1 min each. In a dark environment, grids were incubated with uranyl‐oxalate solution pH = 7 for 5 min, and then placed on ice in contact with a solution of methyl cellulose (9:1) for 10 min. sEVs imaging was obtained using a Tecnai G2 Spirit BioTWIN electron microscope (FEI) at 80 kV.

### 2.7 sEV characterization by Dynamic Light Scattering (DLS)

DLS measurements were done on a Zetasizer Nano ZS (Malvern). The sample was pre‐equilibrated at 37  C for at least 60 s and each measurement was the average of 11 runs. Three consecutive measurements were performed for each sample to evaluate its stability. The results were analysed by the equipment software considering the viscosity and refractive index of water at the measurement temperature, and a refractive index of 1.59 for the scattering particles. The average size was taken from the analysis in volume distribution of particles.

### 2.8 sEV characterization by pulse analysis light scattering (PALS)

NanoBrook ZetaPALS Potential Analyzer (Brookhaven Instruments Corporation, Long Island, U.S.A.) was used for sEV surface charge measurement. Briefly, 5 μl of purified sEVs were diluted in 1500 μl of biological grade ultrapure water (Fisher Scientific, New Hampshire, U.S.A.) and filtered twice through a 0.2 μM filter. sEVs were then placed in a disposable polystyrene cuvette and the electrode was immersed within the cuvette. Each sample was measured five times (using Smoluchowski module) at room temperature.

### 2.9 sEV loading with fluorescently‐labelled miRNA

For the loading of sEVs with a miRNA using the different methods, 10^10^ sEVs were mixed with 10 pmol of miR‐155‐5p‐Cy3 (custom product based on miRIDIAN from Dharmacon modified with 3′end guide strand Cy3) in PBS. To control for miRNA precipitation upon treatment, the miRNA was incubated in the same conditions as described below in the absence of sEVs. Electroporation was carried out in Gene Pulser Xcell™ Electroporation System (Biorad). 10^10^ sEV were resuspended in trehalose pulse medium (50 nM trehalose in PBS), placed in 4 mm cuvettes and pulsed a single time (5 ms) at 400 V. Heat shock was performed in the presence of 0.1 M calcium chloride (Zhang et al., 2016). 10^10^ sEV were placed on ice for 30 min, incubated at 42°C for 1 min and immediately placed on ice for further 5 min. Detergent‐induced membrane permeabilization was performed for 10 min at room temperature in a saponin solution (0.1 mg/ml of saponin in PBS) using 10^10^ sEVs (Fuhrmann et al., 2015). Exo‐Fect loading was carried out by incubating 10^10^ sEV for 10 min at 37°C with Exo‐Fect (10 μl, in a final volume of 150 μl). Cholesterol was also used to complex miRNA with sEVs. In this case, samples were incubated with cholesterol‐modified miRNA (custom product based on miRIDIAN from Dharmacon modified with 5′end passenger strand cholesterol TEG in addition to 3′end guide strand Cy3) for 1 h at 37°C, in a final volume of 100 μl (O'loughlin et al., 2017). Regardless of the method used, all samples were purified using ExoQuick, as per the manufacturer's instructions. Briefly, samples were incubated with Exoquick reagent in 1:5 (v/v) (i.e. 1 ExoQuick volume to 5 sEV sample volumes) for 30 min on ice, centrifuged for 3 min at 13.000 g, the supernatant and the pellet were separated and fluorescence was measured on each fraction.

The emission spectra of all samples, excited at λ_ex_ = 535 nm, was measured from λ_em_ = 563 nm until λ_em_ = 700 nm (incremental steps of 3 nm) in a microplate reader Synergy H1 (Biotek) and the highest point for each sample was considered to calculate the loading efficiency of each method. The loading efficiency on each condition, including the control without sEVs, was calculated using the formula: fluorescence intensity of the pellet/(fluorescence intensity of the pellet + fluorescence intensity of the supernatant). For each condition and each type of sEV, the fluorescence value of the respective control was subtracted to the measured value and this was expressed, in percentage, as the loading efficiency.

For experiments where detection of the miRNA was incompatible with fluorescence, i.e. RT‐qPCR, labelling of miRNA‐124‐Cy5 was used to obtain a fluorescence profile of miRNA‐labelled sEVs. In this case, samples were excited at λ_ex_ = 633 nm, and emission was measured from λ_em_ = 660 nm until λ_em_ = 700 nm (incremental steps of 1 nm).

### 2.10 sEV loading and RNase treatment

mEVs (2 × 10^10^ total particles) were incubated overnight at 4°C with miRNA‐124‐Cy5 (10 pmol) for passive loading, or underwent Exo‐Fect loading as described above. As a control, the same amount of fluorescent miRNA in the absence of sEVs was used. Samples where then purified via ExoQuick as described above in the previous point, and their fluorescence was measured. Subsequently, purified mEV pellets or control pellets were subjected to 2 μg/ml RNAse (# R5125, Sigma‐ Aldrich), in a final volume of 150 μl, treatment for 30 min at room temperature and re‐purified via ExoQuick. Finally, their fluorescence was measured and compared with the results prior to RNAse treatment.

### 2.11 qRT‐PCR analyses of miRNA content

To evaluate miRNA expression in sEVs, total RNA was extracted using the RNeasy Micro Kit (#74004 Qiagen) as per the manufacturer´s instructions. cDNA was synthesized for each sample from the amount of RNA extracted from 2^10^ sEVs using the Mir‐X miRNA First‐Strand Synthesis Kit (#638313, Takara). Finally, qPCR was performed on the CFX Connect Real‐Time System (Bio‐Rad) using the NZYSpeedy qPCR Green Master Mix (2x) (#MB224, Nzytech). Reverse primer was the universal 3′ mRQ primer (Takara). Forward primer sequences were: 5′‐TTAATGCTAATCGTGATAGGGGT‐3′ (hsa‐miR‐155‐5p) and 5′‐GATCTCGTCTGATCTCGGAAG‐3′ (5s rRNA). For RNU6 (RNA, U6 small nuclear) amplification, the forward primer 5′‐TCGGCAGCACATATACTAA‐3′ and the reverse primer 5′‐GAATTTGCGTGTCATCCT‐3′ were used.

### 2.12 sEV dye labelling

Labelling of sEVs with the fluorescent probes 1‐[4‐(trimethylamino)pheny1]‐6‐phenylhexa‐1,3,5‐triene (TMA‐DPH) and N‐hexadecyl‐7‐nitro‐2,1,3‐benzoxadiazol‐4‐amine (NBD‐C_16_) was achieved through the addition of 1% (v/v) from a stock solution of the probe in DMSO, into a solution of sEVs in PBS while gently stirring in the vortex, followed by incubation overnight at 37 °C. For a concentration of sEVs of 8.75 × 10^11^ particles/ml a final concentration of 1 μM TMA‐DPH and 0.1 μM NBD‐C_16_ was used. Loading of sEVs with carboxyfluorescein diacetate succinimidyl ester (CFDA‐SE, #34554 Invitrogen) was performed as per the manufacturer's instructions. Briefly, CFDA‐SE was dissolved in DMSO and sEVs were incubated in a solution of 20 μM of CFDA‐SE in PBS with 2% (v/v) DMSO, for 90 min, at 37 °C. The reaction was stopped by diluting the sample in 0.1% (v/v) BSA in PBS. The sEVs were then attached to CD9 immuno‐labelled magnetic beads (#10620D Invitrogen) as per the manufacturer's protocol. Briefly, beads were washed in PBS and incubated with sEVs overnight at 4°C. Then, samples were washed twice with PBS and the fluorescence of the sEVs was measure on a Cary Eclipse fluorescence spectrophotometer (Varian) equipped with a thermostatted multicell holder. Before the measurements, the sEV solution was transferred to a 5 mm fluorescence cuvette and placed on top of a magnet for 5 min to sediment the sEVs. The cuvette was then transferred to the fluorimeter. The horizontal excitation beam was positioned above the sedimented sEVs thus measuring only fluorescence from CF‐SE in the aqueous supernatant. Fluorescence intensity was followed over time at λ_exc_ = 485 nm λ_em_ = 516 nm for incubation at 37 °C. For in vitro cellular assays, sEVs were labelled with PKH67 (Sigma‐Aldrich) as per the manufacturer´s instructions. Briefly, 2 × 10^10^ sEVs were diluted in the kit buffer (diluent C) 1:1 and then PKH67 in diluent C (1:75) was mixed with the diluted sample. Subsequently, samples were incubated for 3 min at RT, followed by purification by ultracentrifugation as described above. As a control for PKH67 complexation with sEVs, the same protocol, in the applicable assays, was used in the absence of sEVs. In assays where Exo‐Fect‐miRNA was used to modulate sEVs, that step was performed after PKH67 labelling. As a control for that setup, the Exo‐Fect‐miRNA mix was incubated with PKH67 directly and processed was described above.

### 2.13 Exo‐Fect toxicity assays

To assess the cytotoxicity of Exo‐Fect, human umbilical vein endothelial cells (HUVECs) were seeded on 1% (w/v) gelatin‐coated porcine skin (Sigma‐Aldrich) 96‐well plates (Corning), at a density of 10^4^ cells per well in endothelial growth medium 2 (EGM2, Lonza) with EV‐depleted FBS and left to adhere overnight. Cells were either modulated with Exo‐Fect‐miR‐loaded mEVs or native mEVs. Final concentration of miRNA was 25 nM per well. After 24 h, cells were washed with PBS, fixed with 4% (v/v) PFA and washed at RT with PBS. Then, cells were stained with 10 ng/ml Hoechst 33342 for 10 min at RT and imaged using the GE Healthcare InCell 2200 Analyzer imaging system, using a 20× objective, excitation wavelength of 405 nm. Per well, eight different regions of interest were used to count the total number of nuclei and this was used as a proxy for the total number of cells within the different conditions. For the toxicity titration, cells were seeded and handled as detailed above with the exception that in the day following seeding, increasing concentrations of Exo‐Fect, DMSO and ExoQuick were added to the cells and incubated for 24 h. Cells were then fixed and imaged as detailed above.

### 2.14 sEV uptake assay

HUVEC were plated in a 24 well plate at a density of 6 × 10^4^ cells/well and left to adhere for 24 h. Cells were pre‐incubated with different endocytosis inhibitors (details below) for 30 min followed by 4 h co‐incubation with PKH67‐labelled mEVs or Exo‐Fect‐miR‐155‐modulated mEVs (1.5 × 10^9^ particles/ml). The following inhibitors were tested: nocodazole (5 μM), cytochalasin D (25 μM), filipin III (25 μM), chlorpromazine (25 μM) and dynasore (100 μM). The concentrations of the inhibitors were based in values previously reported in the literature (Francia et al., 2018; Paulo et al., 2013) and validated to have no cytotoxic effect during the period of the assay. The toxicity elicited by each inhibitor upon 4.5 h exposure to the cells was evaluated using a CellTiter Glo kit (Promega). After incubation, cells were washed with PBS, trypsinized and centrifuged, followed by 5 min incubation with Trypan blue (0.004% (w/v)) to quench the fluorescence of non‐internalized EVs (Mcneer et al., 2011). Finally, cells were centrifuged, resuspended in PBS and cell fluorescence was quantified by flow cytometry (BD Accuri C6 Plus). As a control, cells were exposed to sEVs in the absence of inhibitors and to inhibit all forms of endocytosis, cells were incubated with sEVs at 4°C.

### 2.15 Intracellular trafficking of sEVs

HUVEC were seeded in a 15 well IBIDI plate at a density of 10^4^ cells/well and left to adhere for 24 h. Cells were incubated with PKH67‐labelled mEVs or Exo‐Fect‐miR‐155‐modulated mEVs (2.5 × 10^9^ particles/ml) for 1, 2 and 4 h in EV‐depleted EGM‐2 medium (Lonza #CC‐3162). After incubation, cells were washed and incubated with LysoTracker red DND‐99 (Invitrogen, 100 nM) for 30 min followed by fixation with 4% (v/v) paraformaldehyde (PFA). To investigate the colocalization with early endosomes, after incubation with sEVs, cells were fixed with 4% (v/v) PFA. Next, cell membrane was stained with a mouse anti‐human CD31 (DAKO, 1:50) primary antibody, followed by incubation with Alexa‐fluor^633^ rabbit anti‐mouse (Invitrogen 1:1000) secondary antibody. In a different subset of experiments, early endosomes were labelled with rabbit anti‐human EEA1 (Cell Signaling Technologies, 1:100) primary antibody followed by incubation with Alexa‐fluor^633^ goat anti‐rabbit secondary antibody (Invitrogen, 1:1000). Cell nuclei were counterstained with DAPI and imaged using the INCell analyzer (GE Healthcare) followed by image analysis using INCell Developer Tollbox. In addition, cells were imaged in a confocal microscope Zeiss LSM 710 to evaluate the colocalization between PKH67‐labeled mEVs and lysotracker. Image acquisition was performed with Plan‐Apochromat 40×/1.4 oil immersion objective and the images were analysed with ImageJ software.

### 2.16 miR functional transfer assay

HEK‐293T transfected with a reporter vector were kindly offered by Dr. Irvin Chen (David Geffen School of Medicine, University of California at Los Angeles). The reporter vector encodes EGFP conjugated to the binding sites of miR‐302a and miR‐302d, and mCherry conjugated to the binding sites of miR‐142‐3p, miR‐155‐5p and miR‐223 (Kamata et al., 2010). HEK‐293T cells were cultured in T‐75 culture flasks (2 million cells/flask) at 37°C in a humidified atmosphere of 5% CO_2_ in DMEM cell culture media containing 10% (v/v) FBS and 0.5% (v/v) penicillin‐streptomycin. For the mCherry knockdown experiments, HEK‐293T cells were seeded in sEV‐depleted medium in collagen‐coated 96‐well plate wells. Cells were left to adhere overnight and the following day, native sEVs (mEVs, uEVs or fEVs) (1.5×10^9^ particles/ml), freshly prepared or stored (> 2 days at ‐80℃) Exo‐Fect‐modulated sEVs (1.5×10^9^ particles/ml), cholesterol‐miR‐modulated mEVs (1.5×10^9^ particles/ml) or Lipofectamine RNAiMAx were used to transfect the cells with miR‐155‐5p or scramble miRNA at a final concentration of 25 nM. As a control for Exo‐Fect‐modulated sEVs, the product of the sEV loading reaction (i.e. Exo‐Fect protocol) performed in the absence of sEVs (fresh or stored) was used in the same proportions. After 24 h, transfection medium was discarded and medium containing 10 ng/ml Hoechst 33342 was added to the cells and after further 48 h medium, without Hoechst 33342, was refreshed. Cells were imaged alive every 24 h after transfection using the GE Healthcare InCell 2200 Analyzer imaging system. The analysis of the images was done using an InCell Investigator package based on the segmentation of the nuclei and quantification of mCherry within the nuclear periphery.

### 2.17 Statistical analyses

All the results showed in this work are presented as an average of the number of samples for each condition and standard deviation (SD). Statistical testing was performed using GraphPad Prism^®^ 6.0 software. The statistical tests used in this work consisted in student's t test and One‐way ANOVA with Dunnet's multiple comparisons test correction. A *P* value < 0.05 was considered statistically significant.

## 3 RESULTS

### 3.1 Exo‐Fect is effective in the loading sEVs with short non‐coding RNAs

**FIGURE 1:**
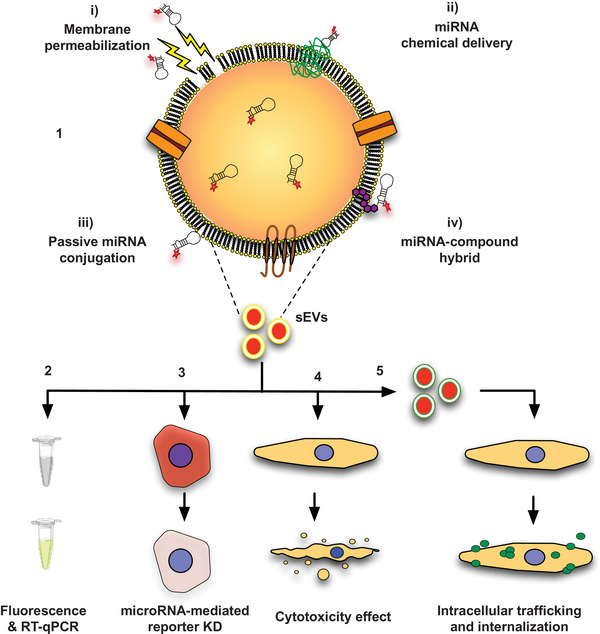
Schematic representation of the different methods used to modulate sEVs with a Cy3‐labelled miRNA mimic and the follow‐up assays performed to validate the modulation and assess the bioactivity of the modulated sEVs. Five different methodologies have been used to load miRNAs into sEVs: transfection by Exo‐Fect or cholesterol‐modified miRNA and membrane permeabilization by a detergent (saponin), electroporation or heat shock. The modified sEVs were then characterized regarding their loading efficiency by fluorescence and qRT‐PCR analyses (Van Niel et al., 2018), bioactivity in the HEK‐293T reporter cell line (Hung & Leonard, 2016), cell toxicity using a cell viability assay (Kanada et al., 2015) and capacity to transfect cells (Montecalvo et al., 2012)

To identify the most efficient method for loading sEVs with a fluorescently‐labelled miRNA, we decided to test, side‐by‐side, five methods previously reported in the literature. Follow‐up experiments were performed to confirm the loading of the miRNA onto the sEVs and their bioactivity (Figure 1). Given the known variability in sEV composition depending on the cell/biofluid source, the most efficient loading strategy was further tested in sEVs isolated from (i) conditioned medium of hUCBMNCs, (ii) human urine and (iii) foetal bovine serum. sEVs secreted from hUCBMNCs (from now on named as mEVs) have been used because these cells are easily obtained from multiple stem cell banks and their regenerative potential in the context of skin wound healing has been recently demonstrated by us (Henriques‐Antunes et al., 2019). sEVs obtained from human urine (uEVs) and bovine serum (fEVs) were used because these fluids are relatively easy to obtain and therefore one can obtain large numbers of sEVs for drug delivery applications. All sEVs were isolated using a standard differential ultracentrifugation protocol (Théry et al., 2006) and characterized by NTA (Supp. Figure 1a), pulse analysis light scattering (PALS) (Supp. Figure 1b) and TEM analyses (Supp. Figure 1c). Regardless of the sEV source, TEM analyses showed the presence of cup‐shaped structures, typical of sEVs. NTA analyses showed that the majority of sEVs had a size in the range of 100–200 nm, which is in accordance with sEVs reported in previous studies (Colombo et al., 2013). In addition, PALS analyses showed that mEVs had a zeta potential of ‐40.2+/‐1.1 mV, while uEVs and fEVs had a zeta potential of ‐18.1+/‐1.5 mV and ‐24.5+/‐1.4 mV, respectively. These differences are likely due to differences in their membrane composition, which ultimately reflect their different origin. As for the purity of our samples, mEVs, uEVs and fEVs showed averages of 2.30 × 10^9^ part/μg, 3.30 × 10^9^ part/μg and 2.60 × 10^9^ part/μg of protein, respectively. Based on previous studies (Webber & Clayton, 2013), our samples fall within the same range of relative low purity, likely owed to the presence of some contaminants, as observed in TEM. To ensure that our preparations were enriched in sEVs, we performed western blot analyses to detect common EV markers and potential contaminants in two different batches of uEVs and mEVs (Supp. Figure 1d). Our results showed that sEVs derived from both sources expressed the markers CD63, CD9 and GAPDH, although their expression level appeared donor‐dependent. Alix was only detectable in uEVs and calnexin, an endoplasmic reticulum marker, was not detected in uEVs. ApoA‐1, a contaminant found in high‐density lipoproteins, was not found in mEVs. Urine sEV samples showed the presence of Tamm‐Horsfall protein (THP), a protein highly present in urine samples (Puhka et al., 2017). Overall, our results showed that our samples were enriched in sEVs.

**FIGURE 2:**
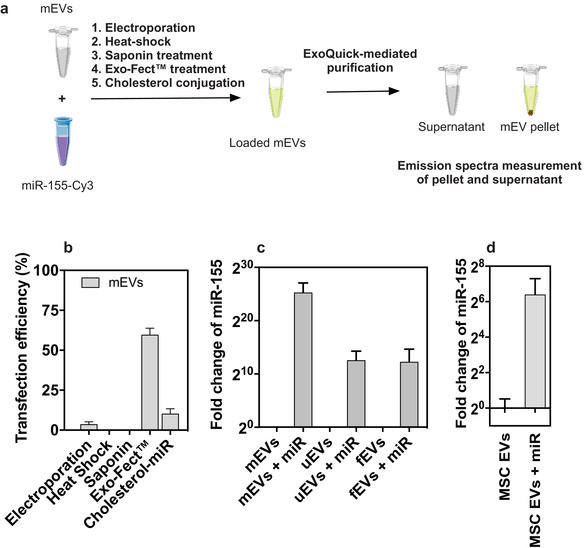
Modulation of sEVs. (a) mEVs were loaded with miRNA‐155‐Cy3 using electroporation, heat shock, saponin permeabilization, Exo‐Fect treatment and cholesterol conjugation. sEVs were then purified with ExoQuick and the fluorescence spectrum of the resulting pellet (sEVs) and supernatant (leftover probe) were quantified. The point of highest fluorescence of each condition was considered for calculating relative transfection efficiencies. (b) Transfection efficiencies were calculated for each condition as described in the Methods section (n = 3 for all conditions tested). (c) qRT‐qPCR analyses of miR‐155‐5p expression in Exo‐Fect‐modulated and native mEVs. Results represent the fold change compared to non‐modulated sEVs. The delta delta Cq method was used for the calculations and 5s RNA was used as a housekeeping control (n = 2‐3 with 2–3 technical replicates). (d) qRT‐qPCR analyses of miR‐155‐5p expression in sEVs derived from MSCs or from MSCs transfected with miR‐155‐5p using lipofectamine RNAiMax (n = 3 with 2 technical replicates)

Next, we evaluated the efficiency of the different methods to load hsa‐miR‐155‐5p‐Cy3 into mEVs. The transfection procedures were based in protocols already published (e.g. electroporation, heat shock, saponin and cholesterol‐modification) (Alvarez‐Erviti et al., 2011; Fuhrmann et al., 2015; Momen‐Heravi et al., 2014; O'loughlin et al., 2017; Zhang et al., 2016) or as per the manufacturer´s instructions (e.g., Exo‐Fect). Importantly, to render the results comparable across the different techniques, the same post‐loading purification method, ExoQuick kit, was used thus yielding two fractions (pellet and supernatant) (Figure 2a). To calculate the loading efficiency, after purification, we quantified the fluorescence of the pellet‐containing sEVs and compared it to the total fluorescence (pellet + supernatant) (Figure 2b). Overall, our results showed that the loading efficiency was higher for sEVs transfected with Exo‐Fect than with the other selected methods (Supp. Figure 2a). In the case of electroporation and heat shock in the presence of calcium chloride, our results suggested that the fluorescently‐labelled miRNA precipitated in the absence of sEVs therefore leading to a sEV‐non‐specific fluorescent signal in the pellet fraction (10% of the total fluorescence for electroporation and 87% of the fluorescence for the heat shock in the presence of calcium chloride) (Supp. Figure 2a). However, in the case of electroporation, after subtracting the fluorescence values of the control, we showed a 3% increase in fluorescence in the pellet fraction. Conversely, in the case of saponin, the vast majority of the fluorescent signal was present in the supernatant fraction, suggesting that it was not possible to load the miRNA into sEVs using this methodology. In the case of Exo‐Fect, our results showed that 50%, 21% and 30% of the fluorescence was found in the pellet fraction of mEVs, uEVs and fEVs, respectively (Supp. Figure 2b), after normalizing to the control. Intriguingly, in the case of Exo‐Fect, we observed an overall decrease in total fluorescence (pellet combined with supernatant) suggesting an Exo‐Fect‐mediated quenching effect, more pronounced in the presence of sEVs, that led to an underestimation of the overall effect of Exo‐Fect (Supp. Figure 2c). In addition, to assess whether the loaded miRNA was exposed or accessible to nucleases after Exo‐Fect transfection, we treated sEVs loaded miR‐124‐Cy5 (through passive loading and Exo‐Fect) with RNAse (Supp. Figure 2d). Our results showed that, in the absence of Exo‐Fect, there was a 73% reduction in the fluorescence of miR‐124‐Cy5, compared to a 11% reduction in fluorescence in the presence of Exo‐Fect. To confirm the loading of sEVs with the exogenous hsa‐miR‐155‐5p, we have quantified by qRT‐PCR the expression of hsa‐miR‐155‐5p on Exo‐Fect‐modulated sEVs from the three different sources (Figure 2c). Our results showed > 2^10^‐fold increase in miR‐155‐5p expression compared to native sEVs. Overall, our results showed that Exo‐Fect was capable of efficiently transfecting sEVs with a miRNA of interest in all the three sEV sources herein tested. The larger differences observed between sEV loading are likely due to differences in the endogenous amounts of the miRNA and housekeeping tested and intrinsic biological properties of sEVs, which, as noted previously, differ, and may render some EV types more easily loadable. However, the fluorescence and miRNA expression patterns were globally similar, with mEVs being the most easily loaded source, followed by fEVs and uEVs.

To confirm that ExoQuick‐mediated purification did not cause co‐precipitation of the labelled miRNA, we performed loading of sEVs with a fluorescent miRNA and Exo‐Fect followed by ODG purification. In total, we obtained 15 fractions (1.5 ml/fraction) of increasing density (Supp. Figure 3a) and per fraction, we quantified the total number of particles and total fluorescence (Supp. Figure 3b). Our results showed that the majority of particles (82%) and fluorescence (73%) localized to fractions 10 to 13 (Supp. Figure 3c), corresponding to the 1.08 g/ml to 1.15 g/ml density range (sEV fraction). In addition, we used qRT‐PCR to quantify the expression of the non‐fluorescent miR‐155‐5p in native and modulated sEVs purified by ODG. Our results showed a > 2^10^‐fold increase in miR‐155‐5p expression in modulated sEVs, a value comparable to the results obtained with the ExoQuick purification (Supp. Figure 3d).

Transfection of EV‐secreting cell with the precursor or mature miRNA has been investigated as a platform to enrich sEVs with a miRNA of interest (Kim et al., 2018). In order to compare post‐isolation modulation with modification of the secreting cell and subsequent harvesting of sEVs, we transfected mesenchymal stromal cells (MSCs) with lipofectamine complexed with a fluorescently labelled miRNA (hsa‐miR‐155‐5p‐Cy3) and isolated the sEVs from the conditioned medium (Figure 2d). Although the fluorescence of sEVs was below the detection limit, we were able to quantify the level of miR‐155‐5p by qRT‐PCR and our results showed a 22‐fold increase in sEVs isolated from transfected MSCs compared to the control (non‐transfected cells) (Figure 2d). However, the concentration of miR‐155‐5p was several orders of magnitude lower than the concentration of miR‐155‐5p observed in sEVs modulated with Exo‐Fect. Based on these results, we decided to investigate in more detail the complex miRNA‐Exo‐Fect‐sEV regarding its biophysical structure and bioactivity.

### 3.2 Exo‐Fect interferes with sEV membrane structure

Currently, it is unknown if Exo‐Fect modulation results in the internalization of the miRNA of interest into the lumen of sEVs or fosters its interaction with the sEV surface. To address this question, we started by characterizing the Exo‐Fect‐modulated mEVs by NTA, TEM and PALS analyses. In the absence of mEVs, Exo‐Fect did not form observable nor quantifiable particles as measured by NTA (Supp. Figure 1) or seen by TEM analysis (Supp. Figure 1). Likewise, in the presence of miRNA, but in the absence of sEVs, no quantifiable particles were detected by NTA (i.e. < 15 particles/frame). However, the Exo‐Fect protocol appeared to induce mEV aggregation as observed by TEM (Supp. Figure 2) and NTA analyses (Supp. Figure 1 and 4c). Data from DLS analysis also supports this hypothesis, showing an increase in the average particle size which correlated with the percentage of Exo‐Fect used with mEVs (Supp. Figure 4d). In addition, the polydispersity of Exo‐Fect‐modulated mEVs increased when higher amounts of Exo‐Fect were used (Supp. Figure 4e). Lastly, ExoQuick‐based purification of mEVs led to a small shift in zeta potential (Supp. Figure 4f), which was further amplified by Exo‐Fect‐mediated transfection of miRNA onto mEVs, from ‐40 mV to ‐20 mV (Supp. Figure 4 g). Collectively, these results suggest that Exo‐Fect may interfere with the membrane of sEVs and ultimately promote their aggregation.

**FIGURE 3:**
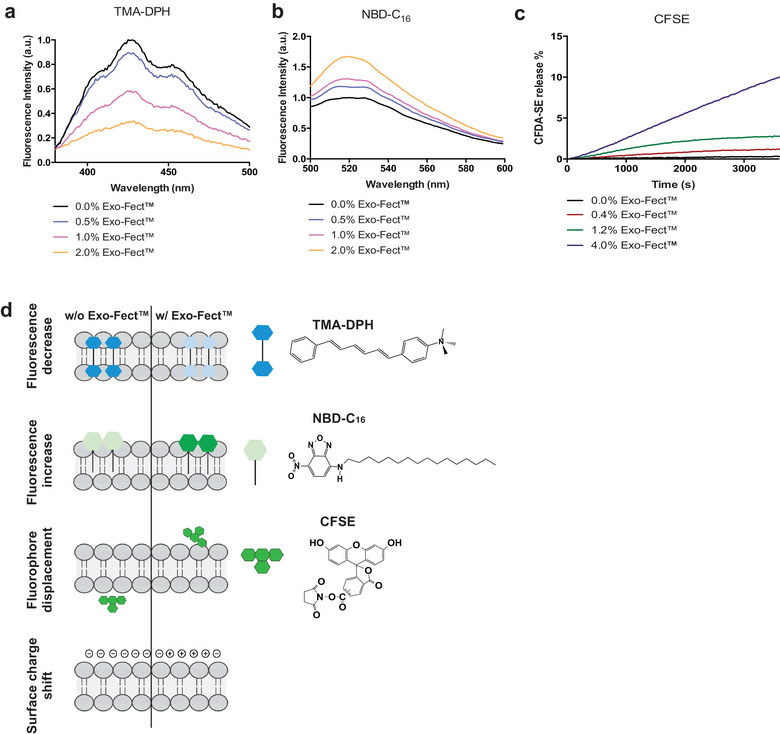
Exo‐Fect interaction with sEV membrane. Effect of increasing amounts of Exo‐Fect on the fluorescence intensity of TMA‐DPH (a) and NBD‐C16 (b), and on the release of encapsulated CF‐SE (b) where the inset shows the fluorescence spectra after 60 min incubation and the main plot shows the release % calculated from the fluorescence intensity increase. The final concentration of Exo‐Fect in (a) and (b) is 0% (□), 0.5% (□), 1% (□) and 2% (□), and in (c) is 0% (□), 0.4% (□), 1.2% (□) and 4% (□). (d) Schematic representation of the proposed mechanism of interaction between Exo‐Fect and sEV membrane regarding how it affects different fluorophores and the surface charge of the sEVs

To confirm that Exo‐Fect interacts with the membrane of sEVs, we performed biophysical analyses in which modulated mEVs were labelled with the fluorescent probes TMA‐DPH or NBD‐C_16_. The fluorescence group of TMA‐DPH is located at the hydrophobic core of the lipid membrane (Do Canto et al., 2016), while the one of NBD‐C_16_ is located at the membrane surface (Amaro et al., 2016; Filipe et al., 2019). The fluorescent probes were equilibrated overnight with the mEVs, leading to a symmetric labelling of both membrane leaflets (Cardoso et al., 2011). The next day, we quantified the changes in fluorescence intensity for both fluorophores in the presence and absence of Exo‐Fect and our results showed that, upon addition of Exo‐Fect to the mEVs labelled with TMA‐DPH, the fluorescence dropped to a third of its initial value (Figure 3a). Conversely, the fluorescence intensity of NBD‐C_16_ increased 3‐fold upon addition of Exo‐Fect (Figure 3b). The observation that both probes were affected by Exo‐Fect suggests that Exo‐Fect alters the properties at the surface as well as in the core of the sEV membrane.

To further confirm that Exo‐Fect interferes with the membrane of sEVs, we encapsulated the fluorescent molecule CFDA‐SE inside mEVs, where it reacts with amino groups from proteins and other biomolecules (Morales‐Kastresana et al., 2017). We conjugated mEVs with anti‐CD9 conjugated magnetic beads in order to isolate mEVs from the solution when required. In the absence of Exo‐Fect, we did not observe a significant increase in the fluorescence of the supernatant after incubation of mEV in PBS during 4 h at 37℃, indicating that there was no significant leakage of encapsulated CFDA‐SE. However, the addition of Exo‐Fect led to an increase in the fluorescence of the supernatant, suggesting that CFSE was leaking from the modulated mEV (Figure 3c). We monitored the increase in fluorescence of the supernatant of these mEVs for several weeks to evaluate the fluorescence signal corresponding to the total leakage of CFDA‐SE (results not shown). Based on this analysis, we were able to calculate the leakage efficiency at the different concentrations of Exo‐Fect tested and we showed that, after only 1 h incubation of mEVs with 2% (v/v) Exo‐Fect at 37℃, 10% of the CFDA‐SE was released from the mEVs. Altogether, these results indicated that Exo‐Fect interacted with the surface of mEVs (Figure 3d) leading to the aggregation and perturbation of its barrier properties. The observation that CFDA‐SE leakage was not instantaneous suggested that for concentrations up to 2% (v/v), Exo‐Fect perturbation of sEV membrane did not lead to a disruption of sEV membrane integrity.

### 3.3 Exo‐Fect allows for functional transfer of miRNA to recipient cells

Having established that Exo‐Fect was an efficient method to modulate sEVs with a miRNA of interest, we decided to evaluate whether Exo‐Fect‐modulated sEVs behaved similarly to their native counterparts in cellular assays. To that end, mEVs were loaded with the miRNA of interest or scramble miRNA (both at 25 nM) using Exo‐Fect as a transfection agent, and administered to human umbilical vein endothelial cells (HUVECs) for 24 h. Our results showed that native mEVs or mEVs modulated with Exo‐Fect at concentrations below 0.5% (v/v) had low (≤10%) impact in cell viability. mEVs modulated with Exo‐Fect at concentrations above 0.5% (v/v) significantly decreased cell viability (Supp. Figure 5a) likely due to the presence of Exo‐Fect. Indeed, Exo‐Fect was toxic for cells in concentrations above 0.5% (v/v) (Supp. Figure 5b). Altogether, our results suggest that sEVs modulated with Exo‐Fect can be used for miRNA delivery with residual cell toxicity for concentrations of Exo‐Fect below 0.5% (v/v), at least in endothelial cells, and this concentration was used for subsequent studies.

**FIGURE 4:**
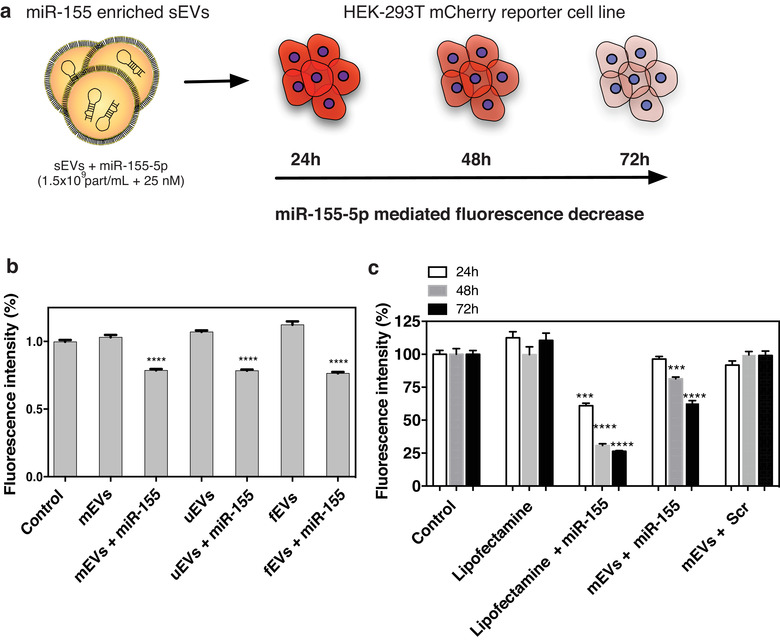
Exo‐Fect‐modulated sEVs are functionally active in vitro. (a) Schematic overview of the protocol used to determine the capacity of miRNA‐modulated sEVs to deliver their cargo onto a HEK‐293T reporter cell line. This reporter cell line constitutively expresses mCherry and contains a binding site for hsa‐miR‐155‐5p on its sequence and thus, upon successfully transfection with miR‐155‐5p, the mCherry signal is reduced proportionally to the transfection efficiency. sEVs (1.5 × 10^9^ part/ml) loaded with miR‐155‐5p or Lipofectamine complexed with miR‐155‐5p was incubated with the reporter cell line (final miR concentration was 25 nM) for 48 h, upon which the medium was changed. After 24 h, the nucleus was stained with Hoechst 33342, the cells were imaged and the fluorescence quantified every 24 h for 3 days. (b) Quantification of the average mCherry fluorescence intensity per cell at 72 h post incubation with mEVs, uEVs, fEVs or their Exo‐Fect‐miR‐155 modulated counterparts. Each condition was normalized to control (HEK‐293T cells with no treatment). Statistical analysis reports to comparisons between each Exo‐Fect‐miR‐155 modulated condition and respective native sEV source. (n = 2‐3) (c) Quantification of the average mCherry fluorescence intensity per cell of native and modulated mEVs and control conditions. Per time point, all conditions were normalized to the control (HEK‐293T cells without treatment). Results were obtained from one experiment with 3 technical replicates. Statistical significance test used was one‐way ANOVA using Dunnet's correction, *P *< 0.05

To evaluate the bioactivity of Exo‐Fect‐modulated mEVs we used a HEK‐293T reporter cell line coding for the mCherry protein, with the target sequence for miR‐155‐5p expressed in its 3′‐UTR (Kamata et al., 2010). Upon successful transfection of this cell line with miR‐155‐5p, the expression of mCherry was downregulated leading to a decrease in the fluorescent signal (Figure 4a). Cells were transfected with Exo‐Fect‐miRNA‐155‐modulated sEVs (mEVs, uEVs or fEVs) or with their native counterparts, for 72 h (Figure 4b) and regardless of the sEV source, the modulation with Exo‐Fect‐miRNA‐155 led to up to 24% decrease in the activity of the HEK‐293T reporter cell line. We next investigated, only with mEVs, whether this effect was time dependent and how it compared with direct transfection of the reporter cell line with lipofectamine, a commonly used transfection agent. In this case, cells were transfected with Exo‐Fect‐miRNA‐155‐modulated mEVs or lipofectamine complexed with the same miRNA and monitored every 24 h for up to 3 days. In cells that were non‐transfected or transfected with lipofectamine alone, the fluorescence did not change. In contrast, cells transfected with miRNA‐155, either with lipofectamine or Exo‐Fect‐miRNA‐155‐modulated mEVs, showed a decrease of 74% and 28%, respectively, in cell fluorescence after 72 h (Figure 4c). Although the efficiency of Exo‐Fect‐miRNA‐155‐modulated mEVs was lower than lipofectamine, the results indicated that mEVs modulated with Exo‐Fect retained their bioactivity.

Next, using the above‐mentioned reporter cell line, we compared the loading efficiency of other methods to Exo‐Fect. To this end, sEVs loaded with cholesterol‐miR‐155, a strategy previously used to load sEVs with miRNAs (O'loughlin et al., 2017), were incubated with the HEK‐293T reporter cell line and our results showed that, compared to the control, no significant change in reporter activity was observed. These results suggest that, under the same testing conditions, this delivery strategy was less efficient (Supp. Figure 6a). The differences observed between our results and previous results may be ascribed to differences in EVs: cholesterol‐miR molecules ratio.

For many applications, the storage of sEVs is required before its use. Therefore, we evaluated whether the biological activity of Exo‐Fect‐modulated sEVs could be compromised by the storage conditions (Maroto et al., 2017). To that end, freshly prepared mEVs were compared with the same batch of modulated mEVs preserved at ‐80℃ for over 2 days. The results showed that the biological activity, assessed using the above‐mentioned reporter cell line, was largely preserved upon storage, with no statistical differences between time points across storage conditions (Supp. Figure 6b). Moreover, in the absence of sEVs, Exo‐Fect‐miR by itself, either used immediately or upon storage at ‐80℃ for over 2 days, was unable to elicit the knockdown of the reporter gene as described above for the formulations containing sEVs (Supp. Figure 6b) supporting the idea that sEVs are crucial for the functional transfer of the miRNA.

**FIGURE 5:**
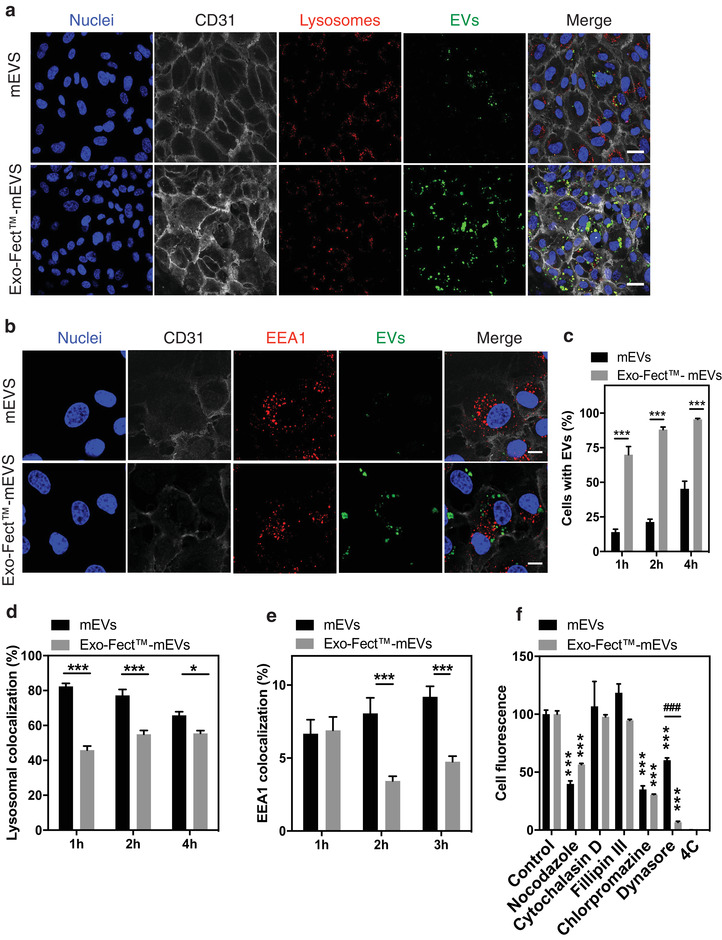
Internalization and intracellular trafficking of Exo‐Fect‐modulated sEVs in endothelial cells. Representative confocal images of HUVECs incubated for 2 h with mEVS (control) and Exo‐Fect‐modulated mEVs, in a colocalization study with lysosomes (a; Lysotracker) and early endosomes (b; EEA1). Scale bar corresponds to 30 μm for lysosomal colocalization images and 10 μm for early endosome colocalization images. (c) Percentage of cells with internalized mEVs and Exo‐Fect‐modulated mEVs as quantified by high content microscopy. (d) Quantification of colocalization with lysosomes and (e) early endosomes. (f) Assessment of internalization routes affected by endocytic pathway inhibitors. HUVEC were pre‐incubated with endocytosis inhibitors for 30 min followed by 4 h co‐incubation of PKH67‐labelled mEVs or Exo‐Fect‐modulated mEVs (1.5 × 10^9^ particles/ml) with each endocytosis inhibitor. After incubation, cells were washed with PBS, trypsinized and centrifuged, followed by 5 min incubation with Trypan blue (0.004% W/V) to quench the fluorescence of non‐internalized sEVs. Cell fluorescence was quantified by flow cytometry. As control, cells were exposed to sEVs without any chemical inhibitor. To inhibit all forms of endocytosis, cells were incubated with sEVs at 4°C. Results are expressed as mean±SEM (in c, d and f n = 3, with 2 technical replicates per experiment; in e, n = 1 with 3 technical replicates). Two‐way ANOVA followed by Bonferroni's post‐test was used to compare mEVs and Exo‐Fect‐modulated mEVs * and *** indicate *P *< 0.05 and *P *< 0.001, respectively. In f, comparison between mEVs and Exo‐Fect‐modulated sEVs, ^###^ indicates *P *< 0.001. Comparison between control and inhibitors, *** indicates *P *< 0.001

Next, we asked whether Exo‐Fect could interfere with the intracellular trafficking of sEVs. To address this question, mEVs were labelled with PKH67, a fluorescent membrane amphiphilic dye commonly used to label sEVs (Kamata et al., 2010; Maas et al., 2015). We confirmed that PKH67 did not fluoresce in the absence of sEVs and that the presence of Exo‐Fect in the sample did not alter sample fluorescence, prior to cell administration (Supp. Figure 7a). Furthermore, Exo‐Fect did not form particles with either PKH67 and/or miRNA that could be localized in the sEV fractions upon purification by ODG (Supp. Figure 7b). After establishing the adequacy of PKH67 to our purposes, HUVECs were incubated with native or Exo‐Fect‐modulated mEVs, for up to 4 h, after which cells were fixated. These cells were subsequently labelled with DAPI (nuclei), CD31 (endothelial cell membrane) and with Lysotracker red (lysosomes – Figure 5a) or EEA1 (early endosomes – Figure 5b). sEV internalization was expressed taking into account the number of cells that had mEVs (green fluorescence) relative to the total number of cells labelled with CD31 (Figure 5c). Approximately 70% of HUVECs internalized Exo‐Fect‐modulated mEVs after 1 h while only 14% of cells internalized native sEVs (Figure 5c). In addition, cells transfected with Exo‐Fect‐modulated mEVs had higher fluorescence than cells transfected with native sEVs indicating that the number of sEVs per cell was higher in Exo‐Fect‐modulated sEVs (Supp. Figure 8a). In order to evaluate whether Exo‐Fect modulation altered sEV intracellular trafficking, we compared the colocalization of mEVs either with lysosomal (Lysotracker^+^) or early endosomal (Early Endosome Antigen (EEA1) 1^+^) compartments. Exo‐Fect‐modulated mEVs had lower co‐localization with the endolysosomal compartment as compared to native sEVs, with a 37% difference at 1 h and a difference of 10% at 4 h (Figure 5d). In addition, Exo‐Fect‐modulated mEVs had also lower co‐localization with early endosomes as compared to native sEVs between 2 and 4 h (2 h: 8% vs. 3.4%; 8 h: 9.2% vs. 4.75%) (Figure 5e). To confirm that the results were not due to differences in the number of lysosomes between the two experimental groups or due to artefacts in the lysotracker staining, we quantified the fluorescence (Supp. Figure 8b) and area of lysosomes per cell (Supp. Figure 8c) with no statistical difference found.

To investigate whether Exo‐Fect played a role in the internalization route of mEVs, HUVECs were pre‐incubated with compounds known to inhibit specific endocytosis pathways (Supp. Figure 8d), namely, nocodazole (microtubule‐dependent endocytosis), cytochalasin D (actin‐dependent endocytosis), filipin III (lipid raft‐dependent endocytosis), chlorpromazine (clathrin‐mediated endocytosis) and dynasore (dynamin‐dependent endocytosis). The concentration of inhibitors used was based in previous studies (Francia et al., 2018; Paulo et al., 2013). Cells were then exposed to PKH67‐labelled mEVs or Exo‐Fect‐modulated mEVs for 4 h, after which their fluorescence was assessed via flow cytometry. Our results showed that cellular uptake of sEVs was mediated by endocytosis, as the cell incubation at 4°C prevented sEV internalization. Moreover, endocytosis inhibition by nocodazole, chlorpromazine or dynasore was effective in reducing sEV uptake (Figure 5f). Interestingly, dynasore was able to inhibit 93% the uptake of Exo‐Fect‐modulated mEVs but only 40% of native mEVs.

## 4 DISCUSSION

Here, we compared side‐by‐side five methodologies to load, post‐isolation, exogenous miRNAs in sEVs obtained from three different sources. The methodology based in the transfection of vesicles with Exo‐Fect yielded the most promising results based in the following parameters: (i) enrichment of miRNAs, (ii) capacity of the modified sEVs to transfer the exogenous miRNA to recipient cells and elicit a biological function (inhibition of the activity of a reporter cell line) and (iii) possibility to store the modified sEVs, for at least 2 days at ‐80℃. Yet, the methodology requires a critical selection of Exo‐Fect concentration for sEV loading to avoid cytotoxicity given the fact that Exo‐Fect remains adsorbed to the membrane of sEVs after purification with Exo‐Quick (the method recommended by the manufacturer). In addition, we showed that Exo‐Fect interferes with the membrane of sEVs.

Previous studies have highlighted the therapeutic potential of sEVs in different pathological contexts. In recent years, a lot of effort has been focused in enhancing the intrinsic potential of sEVs using a plethora of pre‐ and post‐isolation methodologies (Alvarez‐Erviti et al., 2011; Momen‐Heravi et al., 2014; Wahlgren et al., 2012; Yim et al., 2016). Most of the work has been done in loading exogenous biomolecules in sEVs, in particular non‐coding RNAs such as miRNAs (Pomatto et al., 2019). Electroporation has been the most used methodology to load isolated sEVs (Maroto et al., 2017; Momen‐Heravi et al., 2014; Wahlgren et al., 2012); however, the strategy presents important limitations. For example, electroporation may induce miRNA and/or sEV aggregation and, overall, the loading efficiency within the sEVs is very modest (Johnsen et al., 2016; Kooijmans et al., 2013; Lamichhane et al., 2015). In agreement with previous studies, our results indicated that electroporation promoted miRNA precipitation. Other loading strategies based on heat shock in the presence of calcium chloride (Zhang et al., 2016) or the permeabilization of sEV membrane with saponin (Fuhrmann et al., 2015) have been used to load miRNAs into sEVs. According to our results, in the conditions herein tested, around 87% of the miRNA precipitated after heat shock, including in the absence of sEVs. Consequently, we cannot assess how much of that signal might be actual sEV modulation. Conversely, when we used saponin, we could not observe fluorescence in the sEV fraction. When comparing the size and concentration profiles of sEVs before and after treatment with saponin, no difference was found (data not shown), which indicates that sEV stability was not comprised by the detergent. Thus, whether the poor results with both these methodologies were caused by compound interference with ExoQuick remains to be determined and further purification procedures should be tested in future work.

Exo‐Fect was the methodology that resulted in the highest loading of sEVs with an exogenous fluorescently‐labelled miRNA. The loading was monitored using two different methods: (i) fluorescence of the exogenous miRNA loaded in sEVs and (ii) miRNA copies quantified by qRT‐PCR. Different amounts of native miR‐155‐5p within each vesicle source likely contributed to variations in the enrichment of the miR‐155‐5p within each sample. Importantly, the enrichment of sEVs within the miRNA of interest was much higher using this post‐isolation method than the classical transfection of the donor cell with the miRNA of interest followed by the isolation of sEVs from the culture medium. Interestingly, Exo‐Fect methodology decreased the fluorescence of the initial miRNA likely due to a quenching resulting from the high concentration of miRNA loaded in sEVs (Chen & Knutson, 1988). Our results also showed that, depending on the sEV source, the loading efficiency varied which may be explained by the presence of contaminants in some samples. Urine‐derived sEVs contained significant amounts of dark filaments observed by TEM. This is likely THP, a typical protein found in urine which may co‐precipitate with sEVs isolated during ultracentrifugation and found by western blot in our samples (Puhka et al., 2017). Urine contaminants may interfere with different vesicle‐dependent processes (Pisitkun et al., 2004), and that may explain why miRNA‐loading efficiency is reduced for this source of sEVs.

One possible explanation for the results reported herein was related with the possibility that ExoQuick purification could lead to the formation of Exo‐Fect and miRNA complexes that could confound our results. To rule out this, we performed a series of controls where sEVs were absent from the process and showed that while such precipitation may occur (approx. 20%; Supp. Figure 2a), the effect that they may have in functional cellular assays is not measurable using our reporter cell model (Supp. Figure 6b). Nevertheless, the ExoQuick‐based protocol for purification warrants further scrutiny, especially in the context of translational applications. Overall, from a translational standpoint, the methodology presented has some pros and cons. First and foremost, the fact that sEVs may be used from any source post‐isolation, without resorting to donor cell mass production and their respective modification with therapeutic compounds, is an important advantage. Additionally, the fact that the loading protocol is rapid and efficient, potentially capable of complexing different types of nucleic acids with sEVs, renders it a versatile solution. However, the fact that ExoQuick is not the best purification method in terms of sEV yield or purity (Van Deun et al., 2014), leaves space for further improvements to the protocol. Recent discussion has focused on scalable methods to yield high quality sEV preparations in the industrial and clinical scope (Heath et al., 2018). These methods, such as tangential flow filtration and anion exchange chromatography, may be next step towards unlocking the translational potential of sEV formulations.

Our biophysical analyses of sEV modulated with Exo‐Fect lead to a significant decrease in TMA‐DPH fluorescence, which was indicative of a more polar environment around TMA‐DPH (Do Canto et al., 2016). In contrast, the fluorescence of NBD‐C_16_ increased indicating that the polarity around NBD was increased (Amaro et al., 2016). Taken together, these results indicate that Exo‐Fect interacted and changed sEV membrane properties. In addition, Exo‐Fect remained conjugated with sEVs after purification with ExoQuick and this can elicit cytotoxicity above a given concentration (in the case of endothelial cells above 0.5% (v/v)). Moreover, Exo‐Fect presence in sEVs seems to protect the loaded miRNA from RNAse degradation. Further tests are necessary to understand whether the protection is due to the fact that the miRNA is located in the sEV lumen or due to a partial binding of the miRNA to the outer surface of the sEV while Exo‐Fect acts as a protective layer against RNAses.

Functionally, miR‐155 Exo‐Fect‐modulated sEVs were able to inhibit the expression of mCherry in the HEK‐293T reporter line, which, in our construct, had a binding site for this miRNA. While the extent of fluorescence decrease was lower than the one observed by cell transfection mediated by lipofectamine, it remains to be determined whether the limited knockdown effect of modulated sEVs was due to a limited endolysomal escape or a kinetic issue. Moreover, it would be interesting to pursue a similar functional study for all the different methods of sEV modulation, since methods with lower efficiency than Exo‐Fect may still prove to be valuable in a given cellular model and/or therapeutic application. Nonetheless, preliminary tests with cholesterol‐conjugated miR‐155 on sEVs suggest that, under the conditions tested, Exo‐Fect was the most efficient method of miRNA delivery.

Exo‐Fect‐modulated sEVs displayed differences in cell internalization and intracellular trafficking. A previous study has shown that sEVs (without Exo‐Fect modulation) are taken up by cells as single vesicles and a significant portion of sEVs (40%‐60%) seemed to accumulate in lysosomes after several hours and thus their content was likely degraded (Heusermann et al., 2016). Our results showed that 1 h post transfection, sEVs without Exo‐Fect modulation were slowly internalized by endothelial cells (approximately 14% of the cells were labelled with sEVs) but they showed high co‐localization (82%) with the endolysosomal compartment and early endosomal compartments (6.7%). In contrast, within the same time frame, sEVs modulated with Exo‐Fect were rapidly internalized by endothelial cells (approximately 70% of the cells were labelled with sEVs) and showed lower co‐localization (45%) with the lysosomal compartment and similar profiles in endosomal inclusion (6.9% inclusion). At 4 h post transfection, the co‐localization of native sEVs with the lysosomal compartment was still significantly higher than the one of Exo‐Fect‐modulated sEVs (65% vs. 55%, respectively). Likewise, the colocalization with early endosome marker nearly doubled for native sEVs when compared to modulated sEVs (9.2% vs. 4.7% respectively). The lower co‐localization of Exo‐Fect‐modulated sEVs for early time points suggests that modulated sEVs may bypass the endolysosomal compartment more efficiently. Further studies are necessary to elucidate the endolysosomal escape mechanism. In addition, our results seem to indicate an impact of Exo‐Fect on cellular uptake of sEVs. Upon inhibiting endocytosis pathways with different chemical compounds, we have found that both native sEVs and Exo‐Fect‐modulated sEVs were internalized via dynamin and clathrin‐mediated endocytosis given the impact of dynasore and chlorpromazine, as well as nocodazole, a disruptor of microtubules that is also implicated in clathrin‐mediated endocytosis (Jin & Snider, 1993). Specifically, dynasore inhibited the uptake of Exo‐Fect‐modulated mEVs at a higher level than for native mEVs. Dynasore is an inhibitor of dynamin‐mediated membrane fission processes, such as clathrin and caveolae‐dependent endocytosis (Kirchhausen et al., 2008) and our results suggest that these routes of cellular uptake play a larger role for Exo‐Fect‐modulated sEVs than for their native counterparts.

Currently, approximately 30 independent studies have used Exo‐Fect to load sEVs. The majority of these studies focused on loading small RNA duplexes (miRNAs, miRNA inhibitors and siRNAs) (An et al., 2017; Aqil et al., 2019; Castaño et al., 2018; Morton et al., 2018; Zeng et al., 2018) in sEVs whereas others have attempted to load mitochondrial DNA (Ariyoshi et al., 2019), plasmid DNA (Wang & Han, 2019), Y RNA (Cambier et al., 2017) or small peptides (Downs et al., 2018). These reports have established that Exo‐Fect was a viable solution for the complexation of nucleic acids with sEVs. The studies of Pi et al. and Li et al., using a quantification strategy similar to the one herein reported, showed that upon transfection of sEVs with Exo‐Fect, around 80% of the fluorescent signal remained in the sEV fraction of the reaction (Li et al., 2018; Pi et al., 2018). Nevertheless, we added a note of caution when interpreting fluorescent‐based data for calculating the transfection efficiency since Exo‐Fect consistently altered the emission spectra of fluorophores and may also induce a quenching‐like effect. Ultimately, our data supports the idea that Exo‐Fect is an efficient strategy to conjugate small nucleic acids within sEVs and can even enhance the intracellular trafficking and delivery of molecules of interest.

## Supporting information

Supplementary Figure 1. Characterization of sEV isolated from different sources (mEVs, uEVs and fEVs). Samples of mEVs, uEVs and fEVs were analysed via NTA (a), zeta potential (b), and TEM (c). mEVs and uEVs were further analysed by Western Blot (d), where each lane represents a different donor. In all cases n = 2. Supplementary Figure 2. Characterization of sEVs from variable sources modulated by different methodologies. (a) Fluorescence percentage in the pellet fractions of sEVs loaded with miR‐155‐5p‐Cy3. Control indicates that the loading experiment was performed in the absence of sEVs. Results were obtained from 3 independent experiments. (b) Comparison of the transfection efficiency of Exo‐Fect on vesicles isolated from different sources (mEVs, uEVs and fEVs). As a control the same procedure was performed but in the absence of sEVs (shown in white). Results were obtained from 3 independent experiments. (c) Fluorescence measurement of the different stages of sEV modulation with miR‐155‐5p‐Cy3 via Exo‐Fect. Our results showed that immediately after addition of Exo‐Fect to the mixture containing the fluorescently labelled miRNA and sEVs there was a decrease in the overall fluorescence. The majority of that fluorescence was preserved in the pellet (sEV) fraction after purification with ExoQuick. (d) mEVs loaded passively or with Exo‐Fect and miR‐124‐Cy5 were treated with RNase and re‐purified. The loss of fluorescence represents degradation or the miRNA on sEVs or Exo‐Fect, which is markedly lessened by the presence of Exo‐Fect in the reaction. Supplementary Figure 3. Purification and characterization of modulated sEVs by ODG. For the simultaneous detection of miRNA by fluorescence and qRT‐PCR in the same batch of sEVs, sEVs were loaded with both miR‐124‐Cy5 for detection by fluorescence and with miR‐155 for detection by qRT‐PCR analyses. (a) Density of each of the fractions obtained in mEV purification via ODG (n = 3). Relative particle count, as measured by NTA, and relative fluorescence of miR‐124‐Cy5‐labelled mEVs, as measured by fluorometer, of each ODG fraction is shown in (b) and (c) (n = 3). Our results showed that most of the particles localized to fractions 10–13 and that the fluorescence from the labelled miRNA correlated with particle count, indicating that there was a conjugation between sEVs and miR, after Exo‐Fect‐mediated loading. (d) Expression of miR‐155 on fractions 10–13 measured by qRT‐PCR (n = 2). U6 was used as housekeeping gene. Supplementary Figure 4. Characterization of Exo‐Fect‐modulated sEVs. (a) NTA particle size distribution profiles of Exo‐Fect (Raposo & Stoorvogel, 2013) and Exo‐Fect‐modulated sEV (Van Niel et al., 2018). While Exo‐Fect is within background levels, modulated mEVs can only be quantified in sizes generally smaller than 100 nm. (b) TEM images of Exo‐Fect (Raposo & Stoorvogel, 2013) and Exo‐Fect‐modulated mEVs (Van Niel et al., 2018). Exo‐Fect alone was not detected by TEM, but induced visible particle aggregation when complexed with mEVs. (c) NTA profile of Exo‐Fect‐modulated mEVs. Large artefacts obstruct the field of view and mask sample distribution, explaining the results obtained via the quantification. (d) Exo‐Fect‐modulated mEVs show an increase in average particle size, dependent on the Exo‐Fect concentration used (0%, 1%, 2% and 4%). Results are normalized to control (0% Exo‐Fect) and expressed in percentage. This was done because sEVs with high concentrations of Exo‐Fect show high level of aggregation and polydispersity. Results are the average of 3 technical replicates. (e) Polydispersity index of mEVs as measured by DLS, showing an increased heterogeneity dependent on Exo‐Fect concentration. (f) Zeta potential profile of mEVs, mEVs after ExoQuick purification, and (g) mEVs, Exo‐Fect and mEVs complexed with Exo‐Fect, 5, 5, 10, 10 and 5 technical replicates, respectively. Unpaired, two‐tailed t‐test or one‐way ANOVA with Tukey's correction was used to compare all conditions with each other, ** indicates *P* < 0.01 and **** indicates *P* < 0.0001. Supplementary Figure 5. Cytotoxicity of Exo‐Fect‐modulated sEVs against human endothelial cells. (a) Effect of Exo‐Fect‐modulated mEVs on endothelial viability. Endothelial cells were treated with native mEVs or Exo‐Fect‐modulated mEVs. Cell viability was measured by cell counting after 24 h of incubation (n = 1 with 3 technical replicates). Statistical analysis was performed comparing all experimental conditions to untreated control by one‐way ANOVA using Dunnet's correction. **** indicates *P* < 0.0001. (b) Effect of direct administration of Exo‐Fect and ExoQuick on cells (n = 1 with 3 technical replicates). DMSO was used as a positive control for the toxicity assessment based on cell survival. Supplementary Figure 6. Cholesterol‐miR‐modulated sEV efficiency and storage stability of Exo‐Fect‐modulated sEVs. (a) Assessment of the function of cholesterol‐miR‐155‐modulated mEVs on the activity of the HEK‐293T reporter cell line. Quantification of the average mCherry fluorescence intensity per cell at 72 h post incubation with cholesterol‐miR‐155 modulated. The cholesterol‐miR‐155 condition was normalized to control (HEK‐293T cells with no treatment) (n = 1 with 3 technical replicates). Unpaired, two‐tailed t‐test was used to assess statistical significance. (b) Comparison between fresh and frozen (‐80°C for 2 days) Exo‐Fect‐modulated sEVs or Exo‐Fect with miR‐155 on the activity of HEK‐293T reporter. The quantification presented is the average mCherry fluorescence intensity per cell. Stored samples showed no statistical significance when compared to their fresh counterparts for each respective time point. Results are the average of 3 independent runs. Statistical analyses were performed between experimental groups at the same time using a one‐way ANOVA test followed by Dunnet's correction. Supplementary Figure 7. PKH67 interactions with Exo‐Fect. (a) Fluorescence quantification of the same initial amount of native and Exo‐Fect‐modulated mEV samples prior to incubation with HUVECs for internalization experiments. Native sEVs were incubated with PKH67 as described in the methods section. After PKH67 labelling, sEVs were, in relevant conditions, modulated with Exo‐Fect, as described in the methods section. As a control, the same amount of PKH67 was used in solution, in the absence of sEVs. All conditions were purified via ultracentrifugation and their fluorescence was measured by fluorometry. Both samples showed similar levels of fluorescence, while in the absence of sEVs, PKH67 is non‐fluorescent, indicating that its removal from samples was efficient. (b) Quantification of the fluorescence and density of each fraction of an ODG gradient where samples of PKH67 were loaded onto, with and without Exo‐Fect (n = 2), with and without miR‐155‐Cy3. The percentage of PKH67/Cy3 fluorescence relative to total fluorescence after ODG purification was calculated. Supplementary Figure 8. Internalization of Exo‐Fect‐modulated mEVs in HUVECs. (a) Cell fluorescence intensity was quantified after acquisition of images in a high content microscope (INCell analyzer, GE Healthcare) which were then analysed using INCell developer toolbox. (b) Quantification of the area occupied by lysosomes per cell and (c) normalized average intensity of lysosomal probe per cell. (d) Toxicity of each inhibitor used in the internalization studies was assessed after 4.5 h incubation with each inhibitor using CellTiter Glo kit (Promega). Results are expressed as mean±SEM (n = 3, 2 technical replicates for a, b and c, and n = 1 with 2 technical replicates for d).
