# Supplementary material for: Exogenous loading of miRNAs into small extracellular vesicles
Source: J Extracell Vesicles. 2021 Aug 2;10(10):e12111. doi: 10.1002/jev2.12111 (PMC8329988; doi:10.1002/jev2.12111)
Supplement: Supplementary file 1 — Supplementary Figure 1. Characterization of sEV isolated from different sources (mEVs, uEVs and fEVs). Samples of mEVs, uEVs and fEVs were analysed via NTA (a), zeta potential (b), and TEM (c). mEVs and uEVs were further analysed by Western Blot (d), where each lane represents a different donor. In all cases n = 2. Supplementary Figure 2. Characterization of sEVs from variable sources modulated by different methodologies. (a) Fluorescence percentage in the pellet fractions of sEVs loaded with miR‐155‐5p‐Cy3. Control indicates that the loading experiment was performed in the absence of sEVs. Results were obtained from 3 independent experiments. (b) Comparison of the transfection efficiency of Exo‐Fect on vesicles isolated from different sources (mEVs, uEVs and fEVs). As a control the same procedure was performed but in the absence of sEVs (shown in white). Results were obtained from 3 independent experiments. (c) Fluorescence measurement of the different stages of sEV modulation with miR‐155‐5p‐Cy3 via Exo‐Fect. Our results showed that immediately after addition of Exo‐Fect to the mixture containing the fluorescently labelled miRNA and sEVs there was a decrease in the overall fluorescence. The majority of that fluorescence was preserved in the pellet (sEV) fraction after purification with ExoQuick. (d) mEVs loaded passively or with Exo‐Fect and miR‐124‐Cy5 were treated with RNase and re‐purified. The loss of fluorescence represents degradation or the miRNA on sEVs or Exo‐Fect, which is markedly lessened by the presence of Exo‐Fect in the reaction. Supplementary Figure 3. Purification and characterization of modulated sEVs by ODG. For the simultaneous detection of miRNA by fluorescence and qRT‐PCR in the same batch of sEVs, sEVs were loaded with both miR‐124‐Cy5 for detection by fluorescence and with miR‐155 for detection by qRT‐PCR analyses. (a) Density of each of the fractions obtained in mEV purification via ODG (n = 3). Relative particle count, as measured by NTA, [file JEV2-10-e12111-s001.pdf]

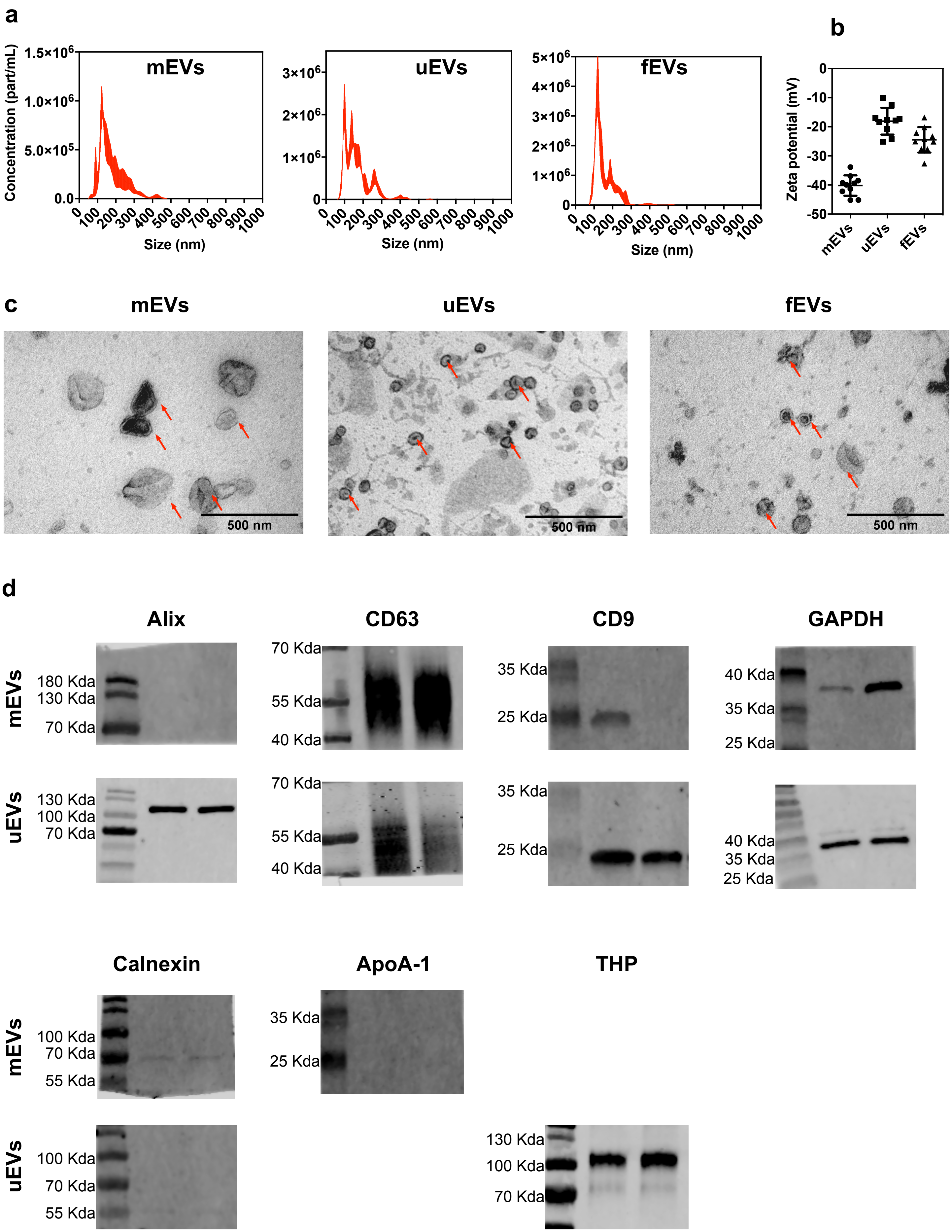
a
mEVs
uEVs
fEVs
Concentration (part/mL)
Size (nm)
b
Zeta potential (mV)
mEVs
uEVs
fEVs
c
mEVs
uEVs
fEVs
500 nm
500 nm
500 nm
d
Alix
CD63
CD9
GAPDH
mEVs
uEVs
180 Kda
130 Kda
70 Kda
130 Kda
100 Kda
70 Kda
70 Kda
55 Kda
40 Kda
35 Kda
25 Kda
40 Kda
35 Kda
25 Kda
Calnexin
ApoA-1
THP
100 Kda
70 Kda
55 Kda
35 Kda
25 Kda
130 Kda
100 Kda
70 Kda

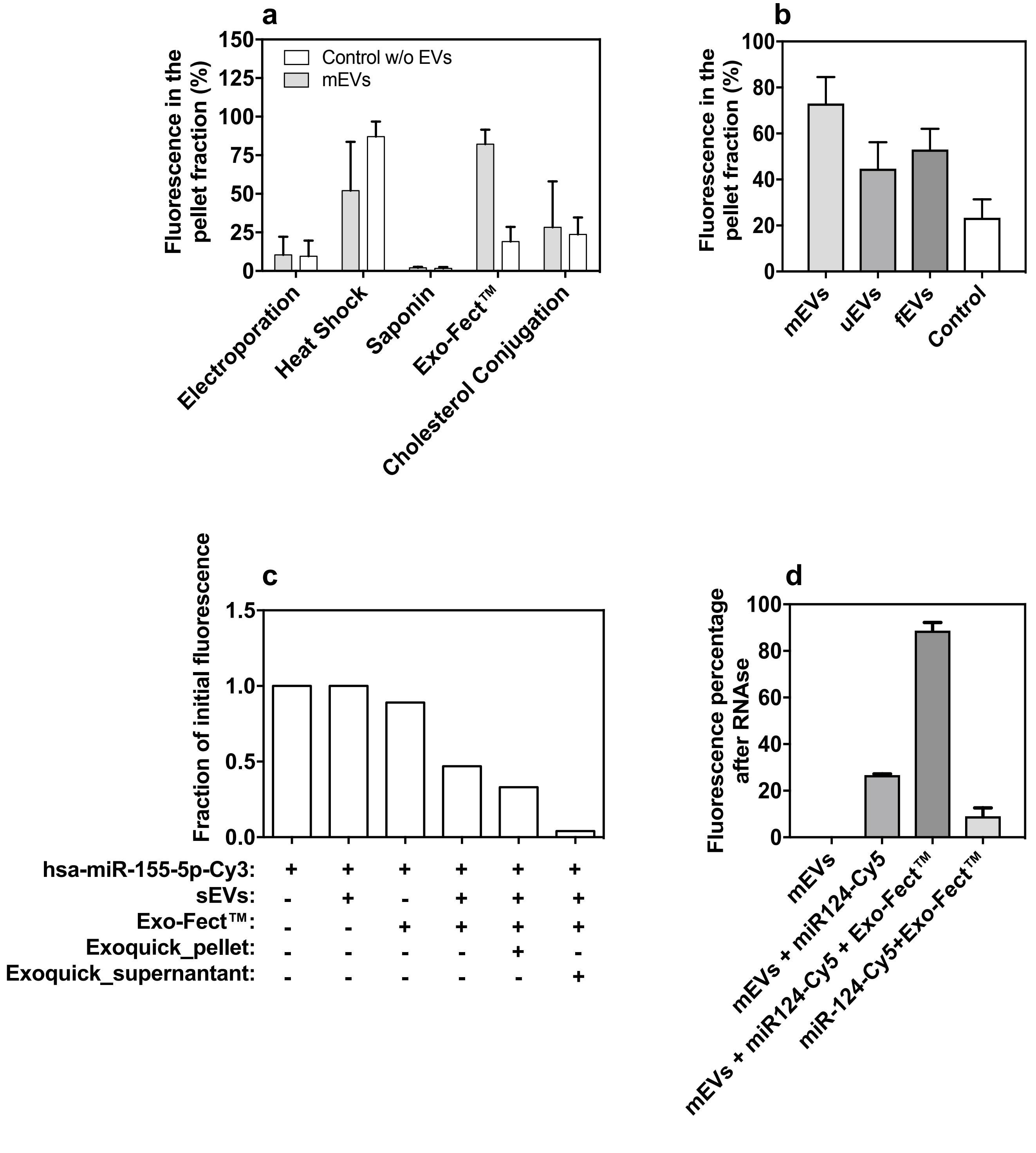

a
Fluorescence in the pellet fraction (%)
150
125
100
75
50
25
0
Control w/o EVs
mEVs
Electroporation
Heat Shock
Saponin
Exo-Fect™
Cholesterol Conjugation
b
Fluorescence in the pellet fraction (%)
100
80
60
40
20
0
mEVs
uEVs
fEVs
Control
c
Fraction of initial fluorescence
1.5
1.0
0.5
0.0
hsa-miR-155-5p-Cy3: + + + + + +
sEVs: - + - + + +
Exo-Fect™: - - + + + +
Exoquick_pellet: - - - - + -
Exoquick_supernantant: - - - - - +
d
Fluorescence percentage after RNAse
100
80
60
40
20
0
mEVs
mEVs + miR124-Cy5
mEVs + miR124-Cy5 + Exo-Fect™
miR-124-Cy5+Exo-Fect™

a

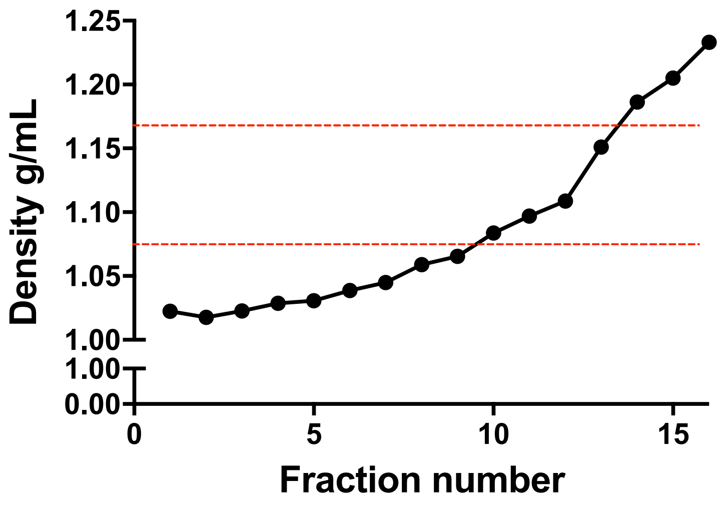

b

Fraction	Relative Particle number	Relative Fluorescence
1	N/A	N/A
2/3	0.32%	5%
4	0.43%	1%
5	0.47%	3%
6	0.47%	2%
7	0.42%	3%
8	0.53%	1%
9	0.94%	5%
10	2.35%	17%
11	29.39%	33%
12	38.59%	15%
13	17.91%	8%
14/15	8.18%	11%

c

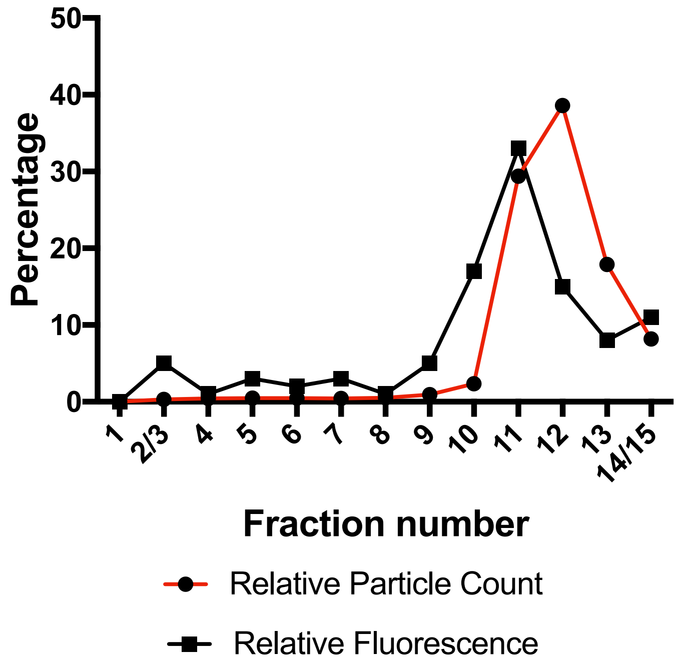

d

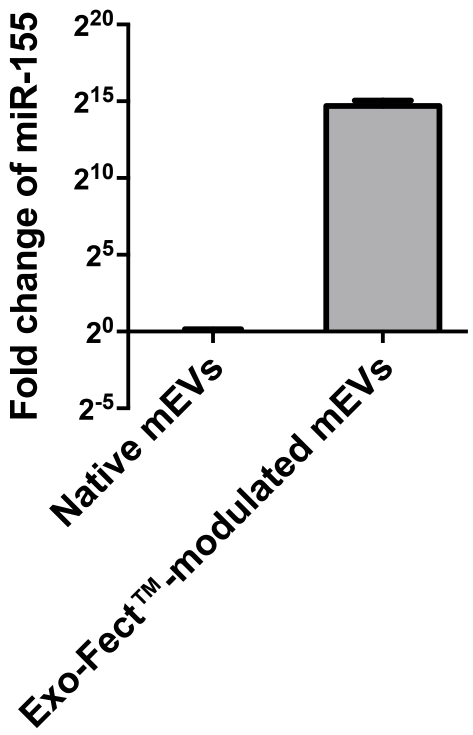

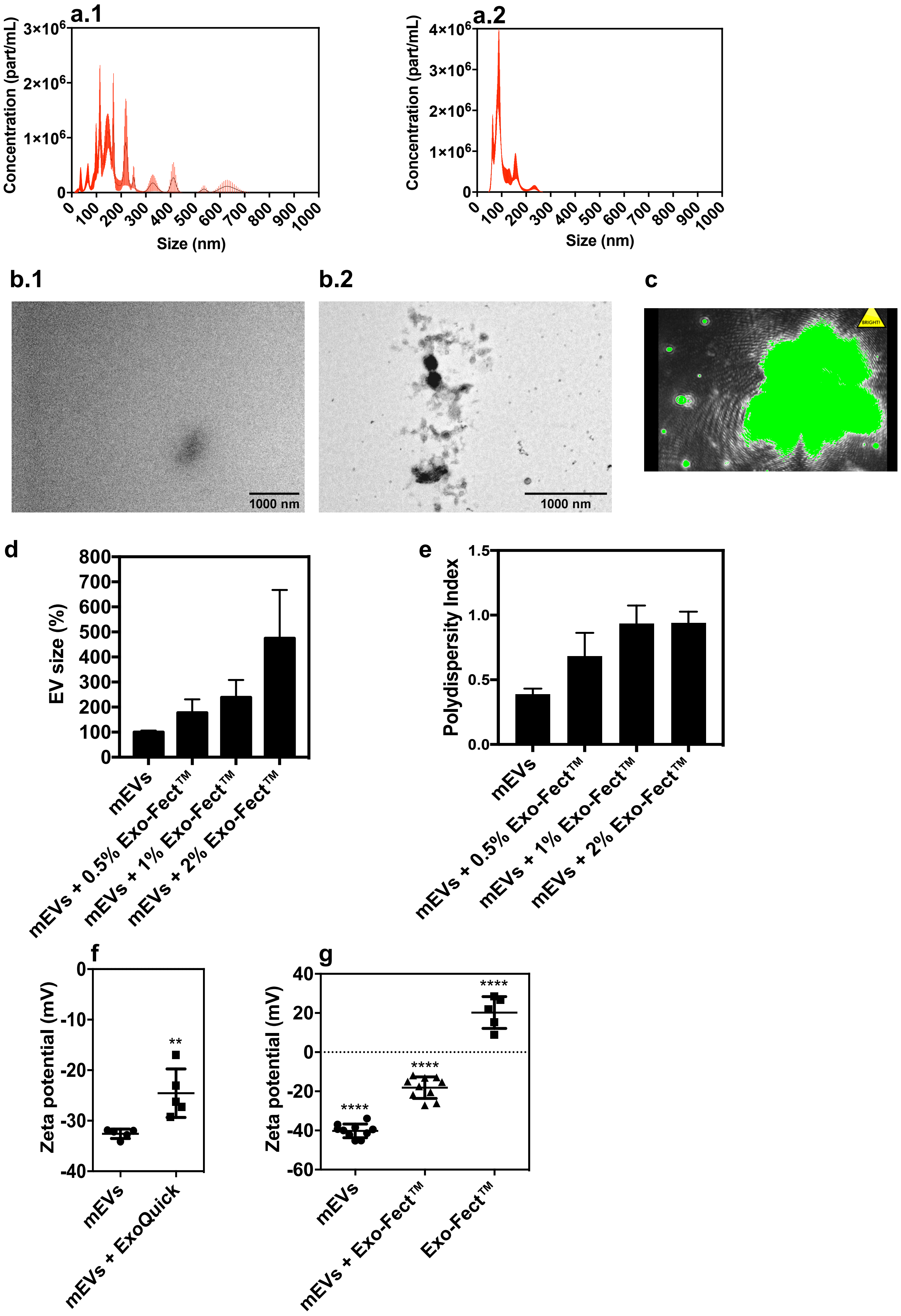

a.1
Concentration (part/mL)
Size (nm)
a.2
b.1
1000 nm
b.2
1000 nm
c
BRIGHT!
d
EV size (%)
mEVs
mEVs + 0.5% Exo-Fect™
mEVs + 1% Exo-Fect™
mEVs + 2% Exo-Fect™
e
Polydispersity Index
f
Zeta potential (mV)
**
mEVs + ExoQuick
g
****
mEVs + Exo-Fect™
Exo-Fect™

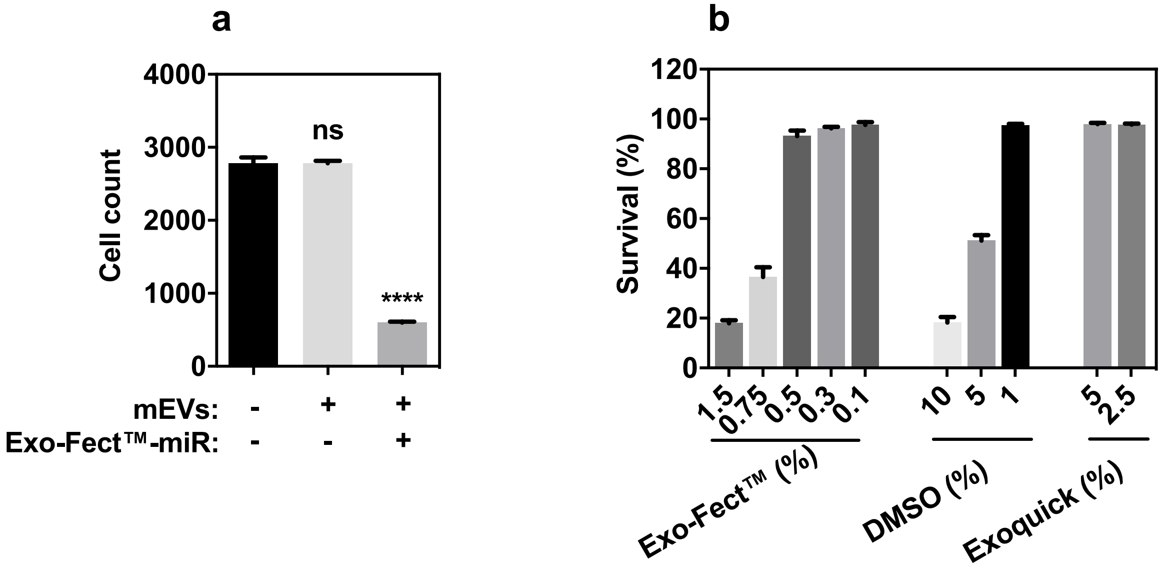
a
Cell count
4000
3000
2000
1000
0
ns
****
mEVs: - + +
Exo-Fect™-miR: - - +
b
Survival (%)
120
100
80
60
40
20
0
1.5
0.75
0.5
0.3
0.1
10
5
1
5
2.5
Exo-Fect™ (%)
DMSO (%)
Exoquick (%)

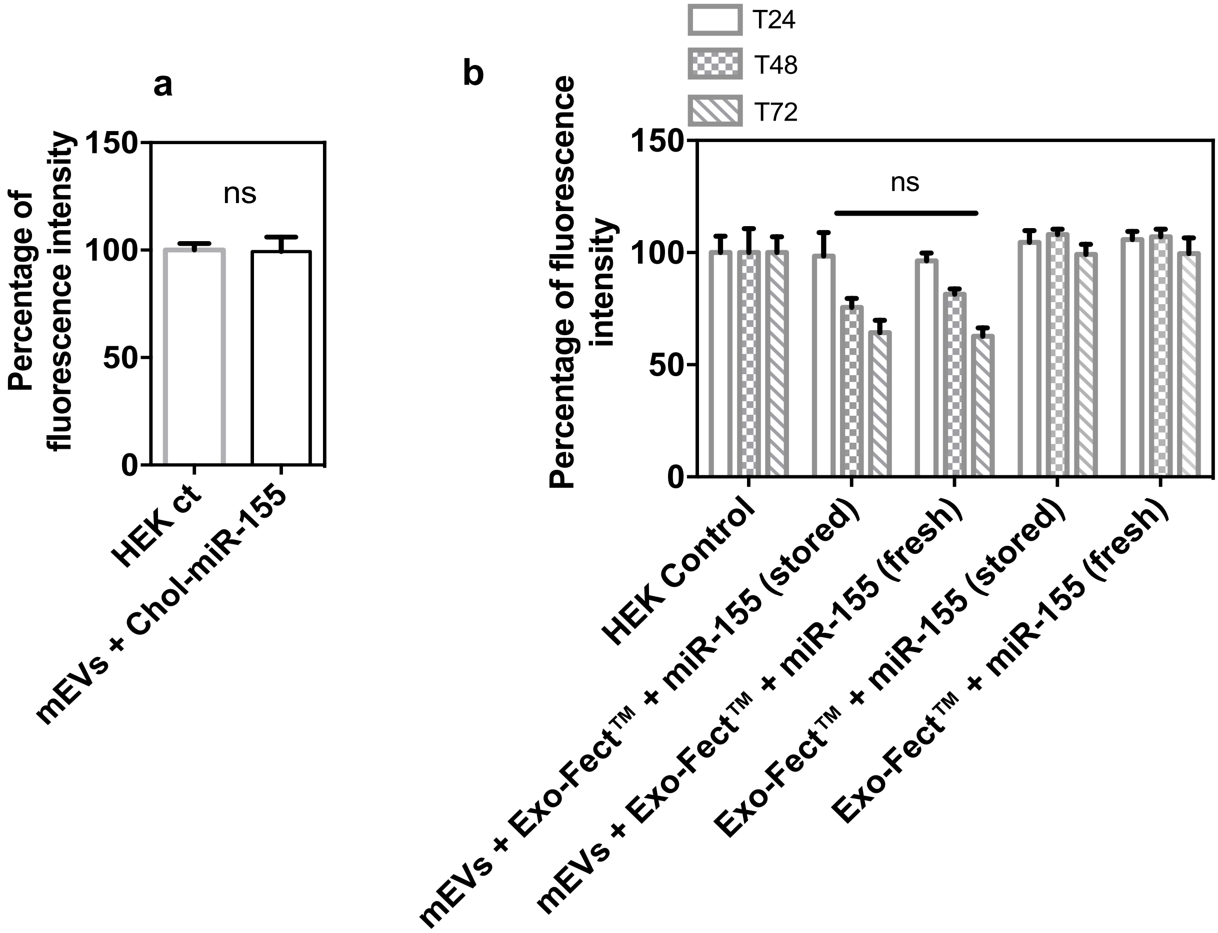
a
b
Percentage of fluorescence intensity
ns
150
100
50
0
HEK ct
mEVs + Chol-miR-155
T24
T48
T72
HEK Control
mEVs + Exo-Fect™ + miR-155 (stored)
mEVs + Exo-Fect™ + miR-155 (fresh)
Exo-Fect™ + miR-155 (stored)
Exo-Fect™ + miR-155 (fresh)

a

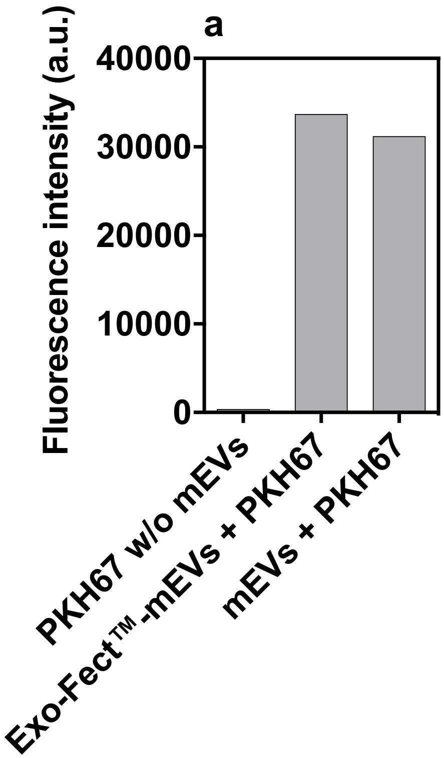

Fluorescence intensity (a.u.)
40000
30000
20000
10000
0
PKH67 w/o mEVs
Exo-Fect™-mEVs + PKH67
mEVs + PKH67

b

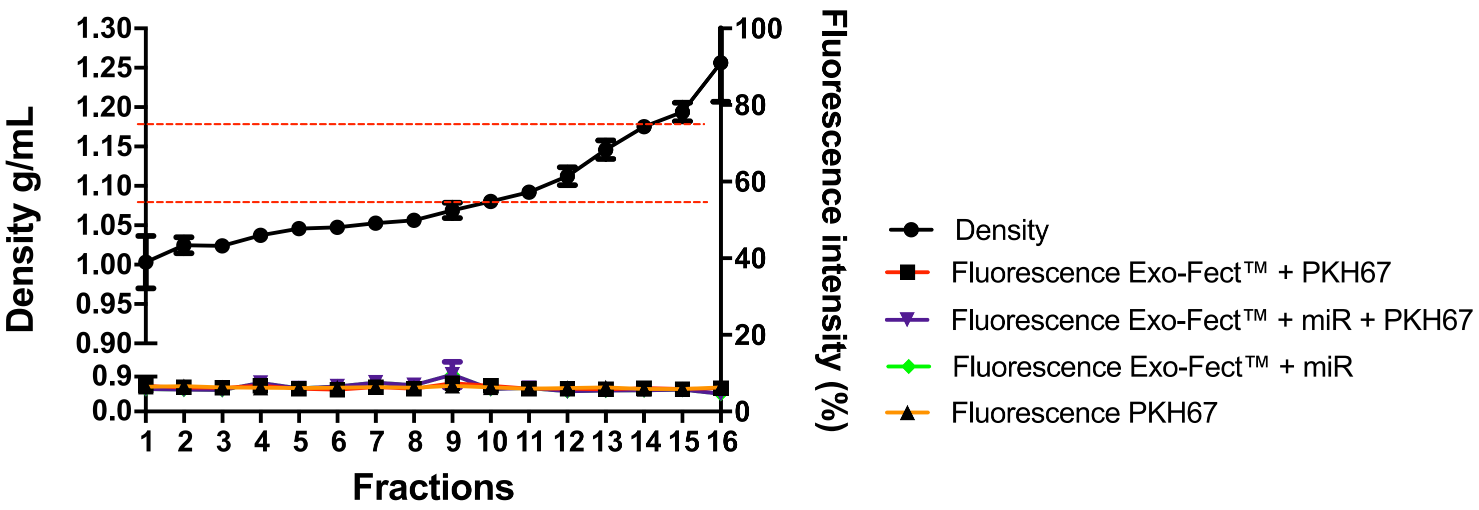

Density g/mL
1.30
1.25
1.20
1.15
1.10
1.05
1.00
0.95
0.90
0.9
0.0
100
80
60
40
20
0
Fluorescence intensity (%)
1 2 3 4 5 6 7 8 9 10 11 12 13 14 15 16
Fractions
Density
Fluorescence Exo-Fect™ + PKH67
Fluorescence Exo-Fect™ + miR + PKH67
Fluorescence Exo-Fect™ + miR
Fluorescence PKH67

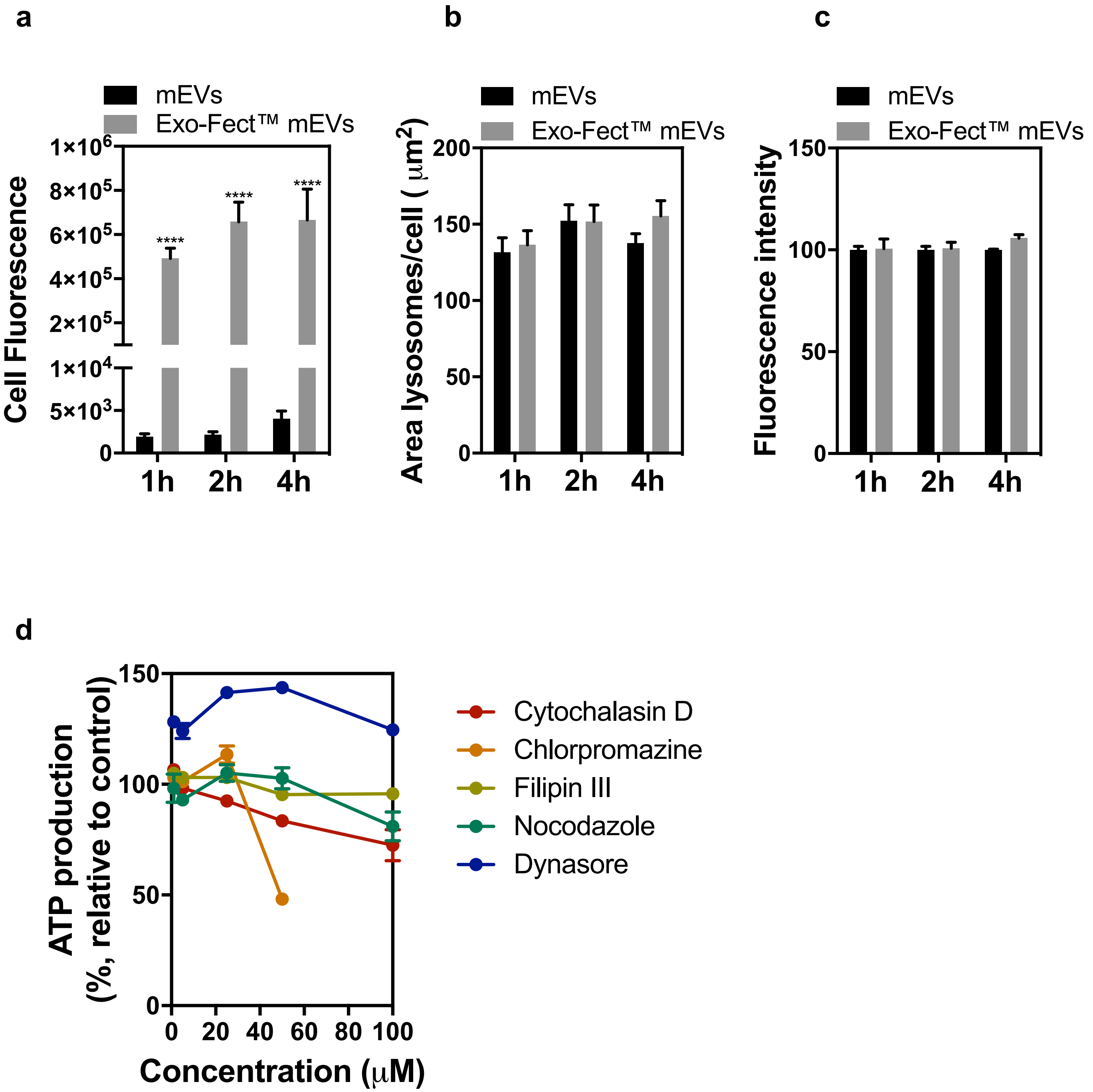
a
mEVs
Exo-Fect™ mEVs
Cell Fluorescence
1×10^6
8×10^5
6×10^5
4×10^5
2×10^5
1×10^4
5×10^3
0
****
****
****
1h
2h
4h
b
mEVs
Exo-Fect™ mEVs
Area lysosomes/cell (μm²)
200
150
100
50
0
1h
2h
4h
c
mEVs
Exo-Fect™ mEVs
Fluorescence intensity
150
100
50
0
1h
2h
4h
d
ATP production
(%, relative to control)
150
100
50
0
0
20
40
60
80
100
Concentration (μM)
Cytochalasin D
Chlorpromazine
Filipin III
Nocodazole
Dynasore
